# C1q-TNF-related protein-3 attenuates pressure overload-induced cardiac hypertrophy by suppressing the p38/CREB pathway and p38-induced ER stress

**DOI:** 10.1038/s41419-019-1749-0

**Published:** 2019-07-08

**Authors:** Bing Zhang, Ping Zhang, Yanzhen Tan, Pan Feng, Zhengbin Zhang, Hongliang Liang, Weixun Duan, Zhenxiao Jin, Xiaowu Wang, Jincheng Liu, Erhe Gao, Shiqiang Yu, Dinghua Yi, Yang Sun, Wei Yi

**Affiliations:** 1 Department of Cardiovascular Surgery, Xijing Hospital, The Fourth Military Medical University, 127 Changle West Road, 710032 Xi’an, China; 2 Department of Geriatrics, Xijing Hospital, The Fourth Military Medical University, 127 Changle West Road, 710032 Xi’an, China; 3 Center for Translational Medicine, Lewis Katz School of Medicine at Temple University, Philadelphia, PA 19140 USA

**Keywords:** Extracellular signalling molecules, Cardiac hypertrophy, Stress signalling

## Abstract

C1q-tumor necrosis factor-related protein-3 (CTRP3) is an adipokine, which exerts protective function in ischemic or diabetic heart injury. However, the role of CTRP3 in cardiac hypertrophy remains unclear. The aim of this study was to investigate the pharmacological effects of CTRP3 on pathological cardiac hypertrophy induced by hypertension. Male C57BL/6 J wild-type (WT) mice, *Ctrp3* knockout mice, and mice infected with lentivirus overexpressing mouse *Ctrp3* underwent sham surgery or transverse aortic constriction (TAC) surgery. After 4 weeks, cardiac hypertrophy, fibrosis, and cardiac function were examined. Compared with WT mice, *Ctrp3* deficiency substantially impaired contractile dysfunction, exacerbated the enlargement of cardiomyocytes and myocardial fibrosis, and reprogramed the expression of pathological genes after TAC. Conversely, CTRP3 overexpression played a role in restoring the left ventricular cardiac contractile function, alleviating cardiac hypertrophy and fibrosis, and inhibiting the expression of hypertrophic and fibrotic signaling in mice after TAC. Furthermore, CTRP3 regulated the expression of the p38/CREB pathway and of the primary modulating factors of the endoplasmic reticulum stress, i.e., GRP78 and the downstream molecules eukaryotic translation inhibition factor 2 submit α, C/EBP homologous protein, and inositol-requiring enzyme-1. Further, inhibition of p38 MAPK by SB203580 blunted the ER stress intensified by *Ctrp3* deficiency. In vitro, CTRP3 protected neonatal rat cardiac myocytes against phenylephrine-induced cardiomyocyte hypertrophy. We conclude that CTRP3 protects the host against pathological cardiac remodeling and left ventricular dysfunction induced by pressure overload largely by inhibiting the p38/CREB pathway and alleviating p38-induced ER stress.

## Introduction

Continuous hypertrophic stimulus such as pressure overload, ischemia, inflammation, and oxidative stress gradually converts the compensatory response into an irreversible pathological cardiac hypertrophy, leading to abnormal expression of the cardiac fetal genes, fibrotic reconstruction of the extracellular matrix, and contractile dysfunction, inescapably progressing to the terminal-stage heart failure^1–3^. Currently, there is no effective drug therapy to reverse the progress from pathological cardiac hypertrophy to heart failure^4^. A widely accepted strategy is positive intervention during the early stage of heart failure, inhibiting the pathological cardiac hypertrophy^5^. Regardless of the continuously developing diagnosis and therapeutic approaches, heart failure has still a poor outcome with nearly 25–50% mortality rate in 5 years after diagnosis^6^. Therefore, seeking out novel effective therapeutic targets to prevent and alleviate the pathological cardiac hypertrophy is urgent and necessary.

The mitogen-activated protein kinase (MAPK) pathway is closely involved in cardiac hypertrophy^7,8^. MAPKs are major regulatory kinases that directly control numerous physiological processes, including cell proliferation, cell growth, and stress responses. Protein 38 MAPK (p38), one branch of MAPKs, can be activated and phosphorylated under pressure-load stimulation^9^. Numerous groups have studied the role of p38 MAPK in various cardiac hypertrophy settings and showed that pathological cardiac hypertrophy can be suppressed by inhibiting the phosphorylation of p38^8,10–12^. The cAMP response element-binding protein (CREB), a downstream signal molecule of p38, can regulate multiple cellular responses by interacting with DNA and regulating gene transcription^13–15^. Various forms of pathological cardiac hypertrophy can be attenuated by inhibiting the phosphorylation of CREB^16,17^.

A variety of stimuli, such as ischemia, hypoxia, free-radical exposure, elevated protein synthesis, and gene mutations, can interfere with the endoplasmic reticulum (ER) function, leading to the accumulation of unfolded and misfolded proteins in the ER. Consequently, multiple ER transmembrane sensors are activated, generating unfolded protein response^18,19^. A long-term nonresolved or excessive ER stress eventually activates the apoptotic signaling pathway. Further, cardiomyocyte apoptosis induced by the ER stress plays an important role in the transition from cardiac hypertrophy to heart failure, and cardiac hypertrophy can be attenuated by alleviating ER stress^18,20,21^. Therefore, ER stress may be an important intervention target to improve pathological myocardial hypertrophy.

C1q/tumor necrosis factor-related protein-3 (CTRP3) is a member of the CTRP superfamily^22,23^. Studies from other groups and our own groups confirmed that CTRP3 exerts a protective effect against myocardial ischemia reperfusion injury, plays a role in the protective response to vascular injury, and can protect the bone marrow mesenchymal stem cells from hypoxic injury^22,24–26^. That is because of the antiapoptotic activity, and vasodilation and angiogenesis effects of CTRP3. Importantly, CTRP3 can alleviate myocardial remodeling and fibrosis in rat after myocardial infarction^27^. Furthermore, CTRP3 attenuates ER stress in mouse with high-fat diet-induced male reproductive dysfunction^28^.

In this study, we investigated the role of CTRP3 in pathological cardiac hypertrophy. CTRP3 deficiency-aggravated cardiac dysfunction, cardiac hypertrophy, and fibrotic remodeling induced by transverse aortic constriction (TAC) in mice. CTRP3 overexpression exerted an opposite effect, and the protective role of CTRP3 was confirmed in vitro. We demonstrate that CTRP3 exerts these effects by inhibiting the p38/CREB signaling pathway and attenuating ER stress. These findings highlight CTRP3 as a therapeutic target for treating pathological myocardial hypertrophy.

## Results

### CTRP3 expression is upregulated in mouse hypertrophic hearts and PE-treated NRCMs

To determine the role of CTRP3 in cardiac hypertrophy, we have examined the mRNA and protein level of CTRP3 in the heart at 1, 2, and 4 weeks after surgery, respectively. After 1 week of TAC, although protein expression level of CTRP3 was slightly elevated, the change was not significant (Fig. S1a). After 2 and 4 weeks of TAC, CTRP3 protein expression level was significantly increased with time after TAC, in parallel with increased levels of the hypertrophic indices including atrial natriuretic peptide (ANP) and myosin heavy chain β (β-MHC) (Fig. S1a). However, the mRNA expression level of CTRP3 was significantly increased at 1, 2, or 4 weeks after TAC compared to SHAM group (Fig. S1b). The mRNA and protein level of CTRP3 in PE-treated NRCMs was also detected. After 24 and 48 h of PE treatment, CTRP3 protein expression was significantly upregulated with time, in parallel with increased levels of ANP and β-MHC (Fig. S1c). Consistently, CTRP3 mRNA expression was also upregulated (Fig. S1d).

### CTRP3 deficiency aggravates cardiac hypertrophy induced by TAC

**Fig. 1:**
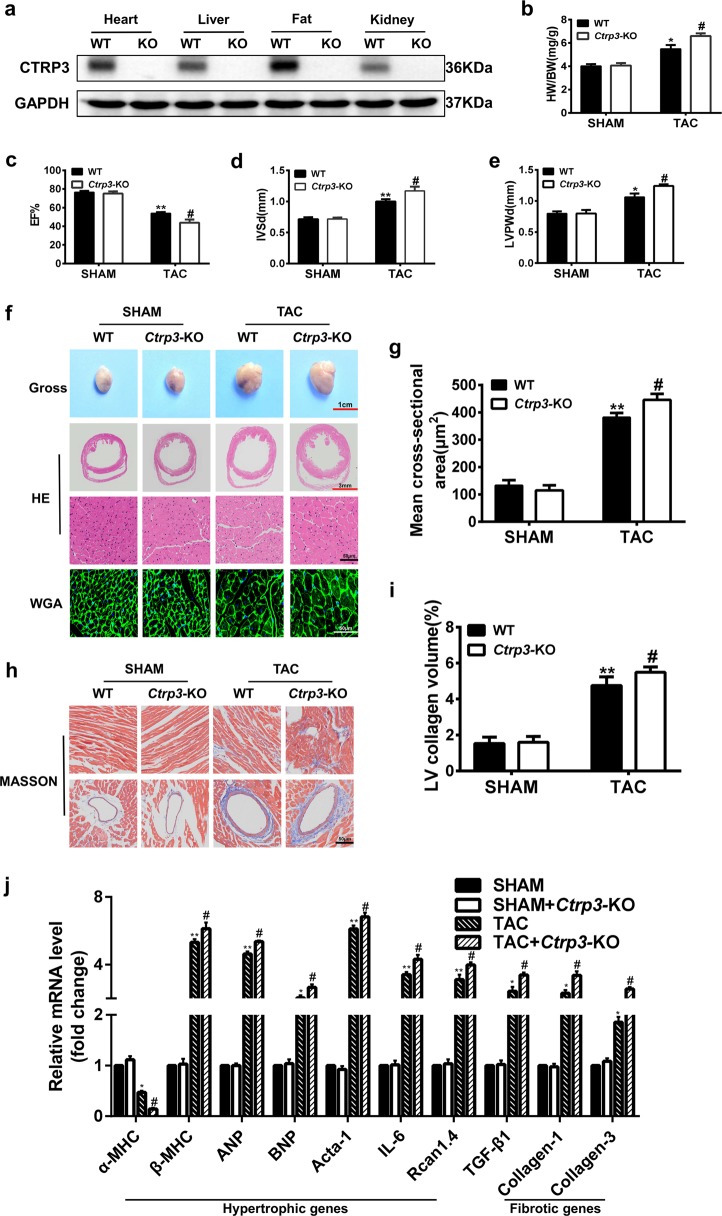
CTRP3 deficiency aggravates cardiac hypertrophy induced by TAC. **a** Representative western blot of CTRP3 levels in different tissues of WT mice and *Ctrp3*-KO mice (*n* = 3 mice per group). **b** The HW/BW ratio in animals after 4 weeks of TAC (*n* = 5–8 mice per group). **c–e** The left ventricular ejection fraction (LVEF), IVSd, and LVPWd, accordingly, determined by analyzing the echocardiographic images (*n* = 12–14 mice per group). **f** Representative images of the gross murine heart and sections stained with hematoxylin and eosin (HE), and wheat germ agglutinin (WGA) (*n* = 5–8 mice per group). **g** The mean cross-sectional area of cardiomyocytes from the indicated groups (*n* ≥ 100 cells per group). **h** Representative images of the murine heart sections (after 4 weeks of TAC) stained with Masson stain, arranged with the perivascular area at the top and the interstitial area at the bottom (*n* = 5–8 mice per group). **i** The LV collagen volume in different groups (*n* ≥ 40 fields per group). **j** Real-time polymerase chain reaction (real-time PCR) analysis of the expression of genes encoding hypertrophic markers α-MHC, β-MHC, ANP, BNP, Acta-1, IL-6, and Rcan1.4, and the fibrotic markers TGF-β1, collagen-I, and collagen-III in each group (*n* = 6 mice per group). **b**–**j** The data were analyzed by one-way ANOVA. **p < 0.01 vs. SHAM, ^#^*p* < 0.05 vs. TAC. In the bar graphs, the data are presented as the mean ± standard error of the mean (SEM)

To evaluate the function of CTRP3 in pressure-overload cardiac hypertrophy, *Ctrp3*-KO mouse was generated (Fig. 1a). No obvious exterior and morphological abnormalities were apparent in 8–10-week-old *Ctrp3*-KO mice compared with the wild-type (WT) mice. Sham or TAC surgery was performed, and 4 weeks later the cardiac hypertrophy model was confirmed by echocardiographic data and histological staining. The heart weight to body weight ratio (HW/BW), and the lung weight to body weight ratio (LW/BW) were higher in *Ctrp3*-KO mice after TAC than in WT mice after TAC (Fig. 1b and Fig. S2a). *Ctrp3*-KO resulted in more severe cardiac dysfunction in TAC-operated mice than in WT mice, as evidenced by decreased ejection fractions (EF)% and fraction shortening (FS)%, and increased interventricular septal thickness at end diastole (IVSd) and left ventricular posterior wall thickness at end diastole (LVPWd) in *Ctrp3*-KO mice subjected to TAC (Fig. 1c–e and Fig. S2b). The representative M-mode echocardiographic images are shown in Fig. S3a. CTRP3 deficiency increased the size of heart in mice after TAC (Fig. 1f). The heart sections were stained with HE, WGA, and Masson stain to analyze the degree of cardiac pathological remodeling. The cardiac hypertrophy and fibrosis of WT mice after TAC were significantly more severe than those in sham-operated WT mice, and CTRP3 deficiency potentiated these responses, as evaluated by determining the average cross-sectional area and left ventricular collagen volume (Fig. 1f–i). Further, after 4 weeks of TAC, the expression of gene for myosin heavy chain α (α-MHC) was significantly downregulated, while that for β-MHC was significantly upregulated in the heart of WT mice compared to sham-operated WT mice (Fig. 1j). CTRP3 deficiency exaggerated this trend (Fig. 1j). The expression of other classic cardiac hypertrophic genes, such as those encoding ANP, brain natriuretic peptide (BNP), α-sarcomeric actin, interleukin 6, and regulator of calcineurin (CaN) 1.4 (Rcan1.4), and fibrotic genes, such as those for the transforming growth factor beta 1 (TGF-β1), collagen-1 (Col-1), and collagen-3 (Col-3) were also upregulated in the cardiac tissue of WT mice subjected to TAC compared to the sham group, while CTRP3 deficiency exaggerated this effect (Fig. 1j). The levels of two major downstream regulatory proteins, CaN and phosphorylated-calmodulin kinase II (CaMKII), were significantly increased in TAC-operated WT mice compared with sham-operated WT mice, and CTRP3 deficiency potentiated this effect (Fig. S4a).

### *Ctrp3* overexpression represses pressure overload-induced cardiac hypertrophy

**Fig. 2:**
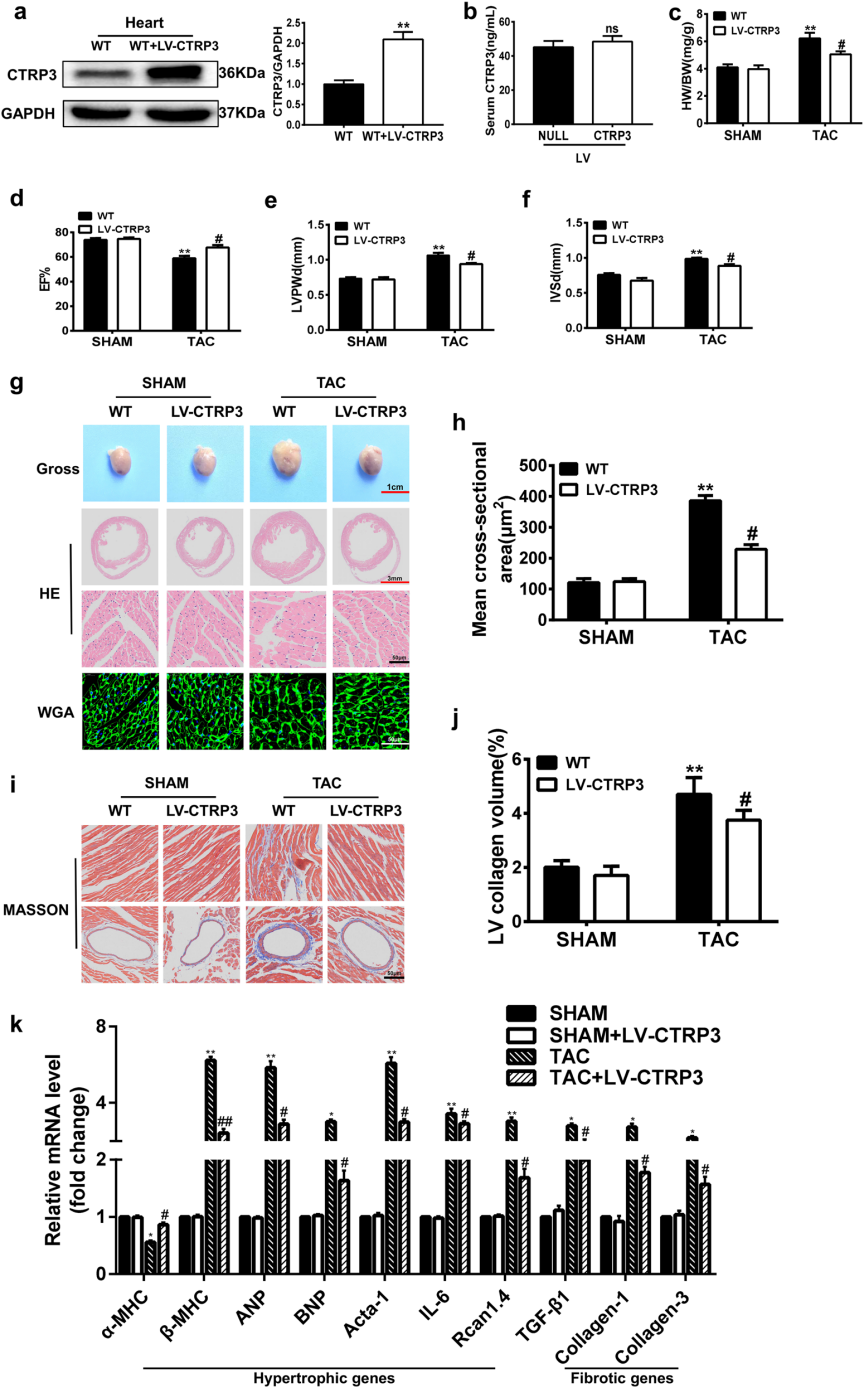
CTRP3 overexpression represses pressure overload-induced cardiac hypertrophy. **a** Representative western blot and quantification of CTRP3 levels in the heart tissue of WT mice and LV-CTRP3 mice (*n* = 3 mice per group). **b** CTRP3 serum levels in mice infected with LV-NULL or LV-CTRP3 (*n* = 3 mice per group). **c** The HW/BW ratio in animals after 4 weeks of TAC surgery (*n* = 5–7 mice per group). **d–f** LVEF, IVSd, and LVPWd, accordingly, determined by analyzing the echocardiographic images (*n* = 12–14 mice per group). **g** Representative images of the gross murine heart and sections stained with HE and WGA (*n* = 5–7 mice per group). **h** The mean cross-sectional area of cardiomyocytes from the indicated groups (*n* ≥ 100 cells per group). **i** Representative images of the murine heart sections (after 4 weeks of TAC) stained with Masson stain, arranged with the perivascular area at the top and the interstitial area at the bottom (*n* = 5–7 mice per group). **j** The LV collagen volume in different groups (*n* ≥ 40 fields per group). **k** Real-time PCR analysis for the expression of genes encoding the hypertrophic markers α-MHC, β-MHC, ANP, BNP, Acta-1, IL-6, and Rcan1.4, and the fibrotic markers TGF-β1, collagen-I, and collagen-III in each group (n = 6 mice per group). **c**–**k** The data were analyzed by one-way ANOVA. *p < 0.05, **p < 0.01 vs. SHAM, ^#^*p* < 0.05 vs. TAC; ns, not significant. In the bar graphs, the data are presented as the mean ± SEM

To confirm the protective role of CTRP3 in pressure-overload cardiac hypertrophy, CTRP3 was overexpressed in the cardiac tissue by injection of a lentivirus harboring the *Ctrp3* gene (LV-CTRP3) (Fig. 2a). However, as determined by enzyme-linked immunosorbent assay (ELISA), *Ctrp3* overexpression in the myocardial tissue was not sufficient to cause a significant increase in serum CTRP3 levels (Fig. 2b). There are no obvious exterior and morphological differences between 8–10-week-old sham-operated WT mice and LV-CTRP3 mice (data not shown). No morphological and functional changes of the heart were observed at baseline, but *Ctrp3* overexpression significantly reversed TAC-induced decrease in the HW/BW and LW/BW, indicating a relief of pulmonary congestion (Fig. 2c and Fig. S2c). Moreover, the above-mentioned echocardiography-detectable dysfunction and ventricular dimension enlargement were attenuated in TAC-operated LV-CTRP3 mice compared with WT mice after TAC (Fig. 2d–f, Figs. S2d, S3b). Analysis of the gross heart morphology indicated that *Ctrp3* overexpression reduced the heart size in mice after TAC (Fig. 2g). In addition, TAC-triggered cardiac hypertrophic and fibrotic remodeling were blunted in TAC-operated LV-CTRP3 mice compared with WT mice after TAC, as evidenced by reduced average cross-sectional area and left ventricular collagen volume (Fig. 2g–j). Finally, *Ctrp3* overexpression reversed the expression trend of α-MHC and β-MHC genes, and decreased the degree of upregulation of the above-mentioned marker genes in TAC-operated mice (Fig. 2k). *Ctrp3* overexpression also alleviated the activation of CaN and CaMKII (Fig. S4b).

### CTRP3 alters phenylephrine (PE)-induced cardiomyocyte hypertrophy in neonatal rat cardiac myocytes (NRCMS)

**Fig. 3:**
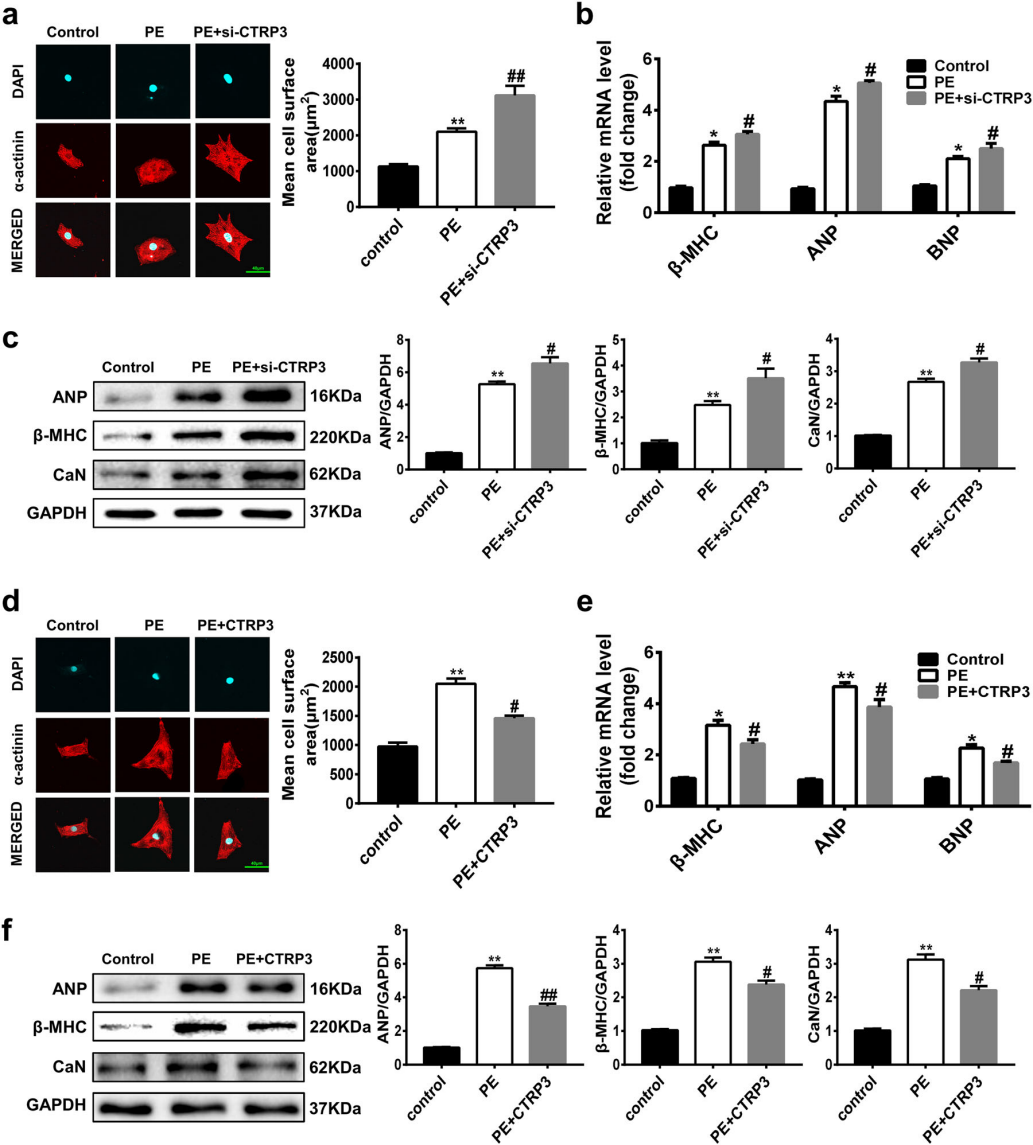
CTRP3 alters PE-induced cardiomyocyte hypertrophy in NRCMS. **a** Left: Representative immunofluorescence images of the NRCMs stained by α-actinin (red) and 4′,6-diamidino-2-phenylindole (DAPI; blue) observed under a confocal microscope. Right: The mean cell surface area of NRCMs from the indicated groups (*n* ≥ 30 cells per group). **b** Real-time PCR analysis of the expression of genes encoding the hypertrophic markers β-MHC, ANP, and BNP in the indicated groups (*n* = 5 samples per group). **c** Representative western blot (left), and quantification (right) of β-MHC, ANP and CaN levels in the indicated groups (*n* = 5 samples per group). **d** Left: Representative immunofluorescence images of the NRCMs stained by α-actinin (red) and DAPI (blue), observed under a confocal microscope. Right: The mean cell surface area of NRCMs from the indicated groups (*n* ≥ 30 cells per group). **e** Real-time RT-PCR analysis of the expression of genes encoding the hypertrophic markers β-MHC, ANP, and BNP in the indicated groups (*n* = 5 samples per group). **f** Representative western blot (left), and quantification (right) of β-MHC, ANP and CaN levels in the indicated groups (*n* = 5 samples per group). The data were analyzed by one-way ANOVA. *p < 0.05, **p < 0.01 vs. control, ^#^*p* < 0.05 vs. PE. In the bar graphs, the data are presented as the mean ± SEM

To further test the antihypertrophic effect of CTRP3, a PE-induced cardiomyocyte hypertrophy model was established. PE-treated NRCMs were administered CTRP3 or *Ctrp3* siRNA (si-CTRP3). The knockdown effect of si-CTRP3 on CTRP3 expression was detected and significant (Fig. S5). We found that CTRP3 depletion exacerbated the PE-induced increase of cell size, and mRNA level of β-MHC, ANP, and BNP (Fig. 3a, b). Changes in ANP, β-MHC, and CaN protein levels were consistent with the immunostaining and gene expression data (Fig. 3c). However, these trends were completely reversed by CTRP3 supplementation, indicating a protective role of CTRP3 against pathological cell growth in vitro (Fig. 3d–f).

### CTRP3 regulates activation of the p38 MAPK/CREB pathway in TAC mouse

**Fig. 4:**
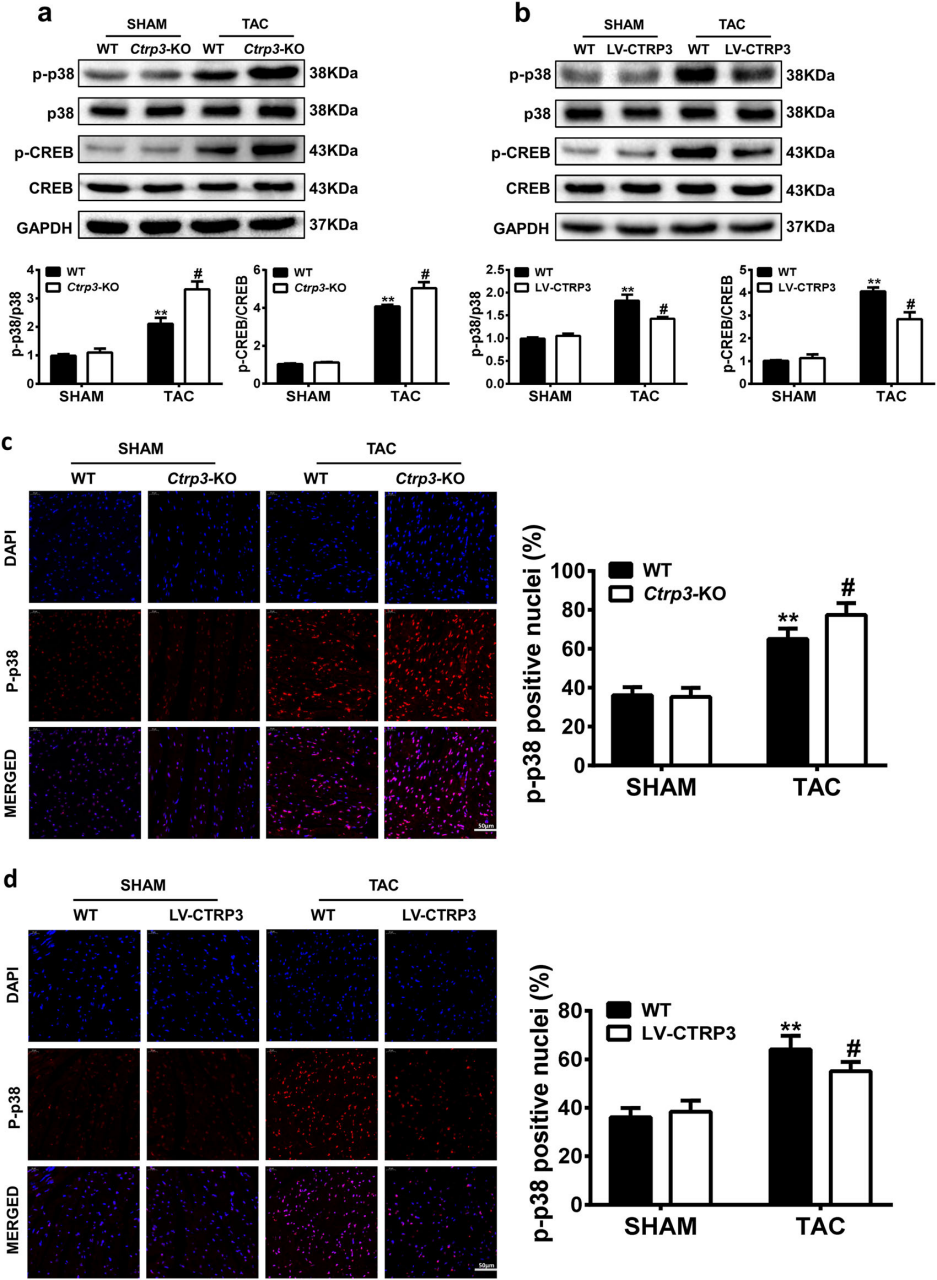
CTRP3 regulates activation of the p38 MAPK/CREB pathway in TAC mouse. **a, b** Representative western blot (top) and quantification (bottom) of the p38-CREB signaling pathway activity in the hearts of mice with different genotypes (WT, *Ctrp3*-KO, and LV-CTRP3) 4 weeks after sham treatment or TAC surgery (*n* = 5–6 mice per group). **c, d** Representative immunofluorescence images of murine heart sections stained with p-p38 (red) and DAPI (blue) (left), and the percentage of p-p38–positive nuclei (right) in the hearts of mice with different genotypes (WT, *Ctrp3*-KO, and LV-CTRP3) 4 weeks after sham treatment or TAC surgery (*n* = 5–6 mice per group). The data were analyzed by one-way ANOVA. *p < 0.05, **p < 0.01 vs. SHAM, ^#^*p* < 0.05 vs. TAC. In the bar graphs, the data are presented as the mean ± SEM

To explore the mechanism of CTRP3-based inhibition of cardiac hypertrophy, several signaling pathways related to the process of hypertrophy were tested. We found that the phosphorylation and expression levels of AMP-activated protein kinase (AMPK) and Akt were not affected by changes in CTRP3 expression (Fig. S6). However, after 4 weeks of TAC, the phosphorylation of p38 MAPK and its downstream target CREB were increased, and this effect was significantly potentiated in TAC-operated *Ctrp3*-KO mice (Fig. 4a). The upregulation of p38 and CREB phosphorylation after TAC was significantly reduced in LV-CTRP3 mice (Fig. 4b). The immunofluorescence intensity of p-p38 was stronger in TAC-operated WT mice than in sham-operated WT mice, and CTRP3 deficiency enhanced this change (Fig. 4c). The immunofluorescence intensity of p-p38 in TAC mice was also decreased by *Ctrp3* overexpression (Fig. 4d).

### Protein p38 MAPK activation mediates give-and-take of CTRP3-regulated cardiac hypertrophy and fibrosis

**Fig. 5:**
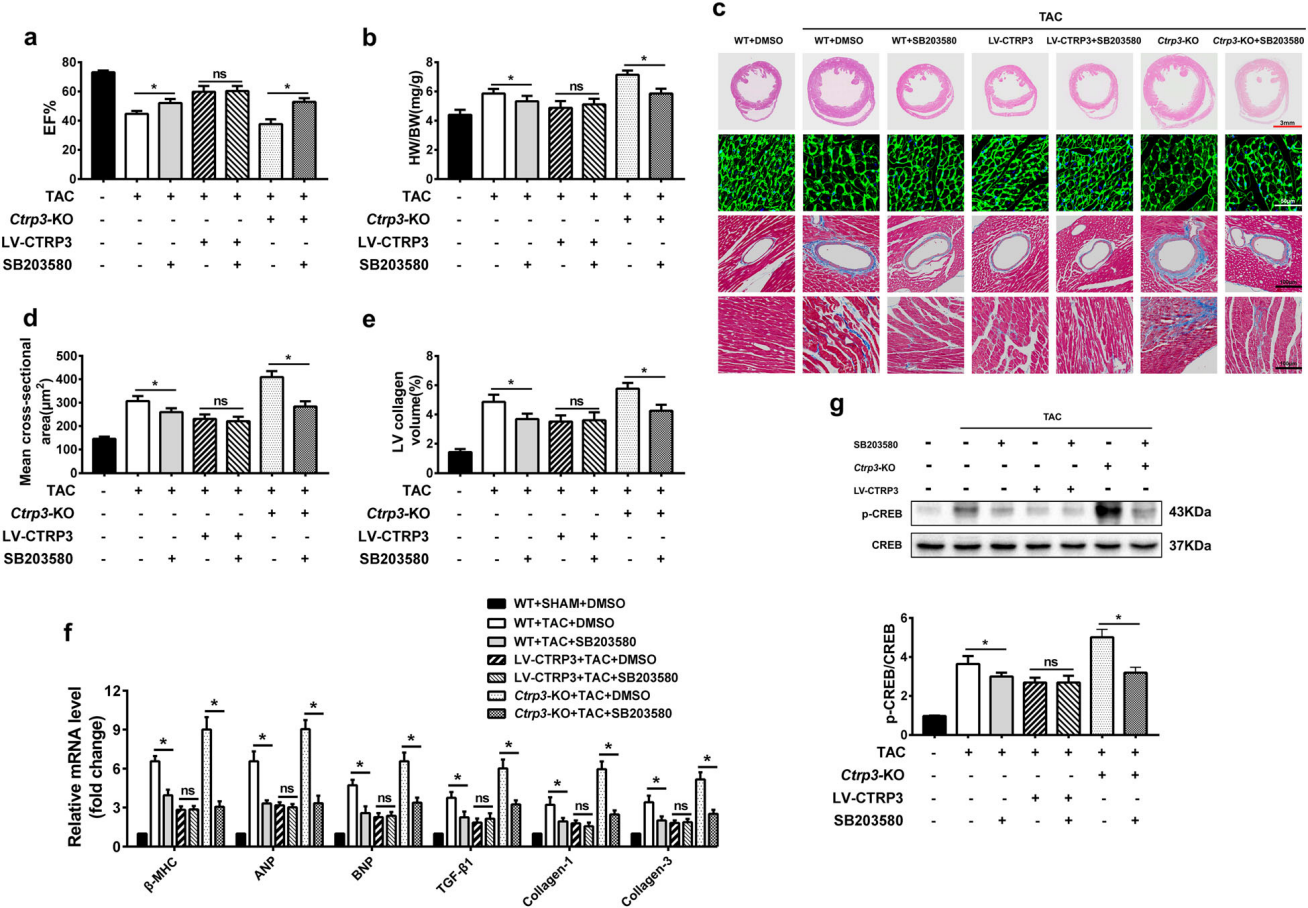
P38 MAPK activation mediates CTRP3 deficiency-aggravated cardiac hypertrophy and fibrosis. **a** Ejection fraction (EF)% after 4 weeks of TAC in the indicated groups (*n* = 5–6 mice per group). **b** The HW/BW ratio in animals from indicated groups after 4 weeks of TAC (*n* = 5–6 mice per group). **c** Representative images of the heart sections stained with HE, WGA, and Masson stain (*n* = 5 mice per group). **d** The mean cross-sectional area of cardiomyocytes from the indicated groups (*n* ≥ 100 cells per group). **e** The LV collagen volume in different groups (*n* ≥ 40 fields per group). **f** Real-time PCR analysis of the expression of genes encoding the hypertrophic markers β-MHC, ANP, and BNP, and the fibrotic markers TGF-β1, collagen-I, and collagen-III in each group (*n* = 5 mice per group). **g** Representative western blot (top) and quantification (bottom) of the CREB activity in the hearts of mice from indicated groups (*n* = 5 mice per group). The data were analyzed by one-way ANOVA. *p < 0.05 between the two indicated groups; ns, not significant. In the bar graphs, the data are presented as the mean ± SEM

To further explore the role of CTRP3 in regulating the p38 MAPK signaling pathways in TAC-operated mice, SB203580 was used to inhibit the activation of p38 MAPK. After SB203580 treatment, EF%, HW/BW, ventricular wall thickness, cross-sectional area, and left ventricular collagen volume were markedly improved in mice from WT + TAC + SB203580 group compared to WT + TAC + DMSO group (Fig. 5). After TAC, some LV-CTRP3 mice were treated with SB203580. No significant differences in EF%, HW/BW, ventricular wall thickness, cross-sectional area, and left ventricular collagen volume were apparent in the presence or absence of SB203580 in LV-CTRP3 mice after TAC (Fig. 5). This indicated that the inhibition of p38 did not affect the antihypertrophic role of CTRP3. When *Ctrp3*-KO mice were treated with SB203580 after TAC, the cardiac hypertrophy (Fig. 5c, d) and fibrosis (Fig. 5c, e) aggravated by CTRP3 deficiency were significantly improved, as well as the damage of cardiac function (Fig. 5a) and increased HW/BW (Fig. 5b). The expression of hypertrophic and fibrotic marker molecules was consistent with the phenotypic observations (Fig. 5f). Moreover, p38 inhibitor treatment significantly decreased the TAC-induced activation of CREB in heart. Further aggravated CREB activation in *Ctrp3-*KO mice was also decreased by p38 inhibitor. And there was no significant difference of CREB activation between TAC + LV-CTRP3 + DMSO group and TAC + LV-CTRP3 + SB203580 group. (Fig. 5g). Collectively, these findings suggested that CTRP3 inhibited pathological cardiac hypertrophy at least by inhibiting the activation of the p38/CREB signaling cascade.

### CTRP3 alleviates PE-induced cardiomyocyte hypertrophy in vitro by inhibiting the p38/CREB pathway

**Fig. 6:**
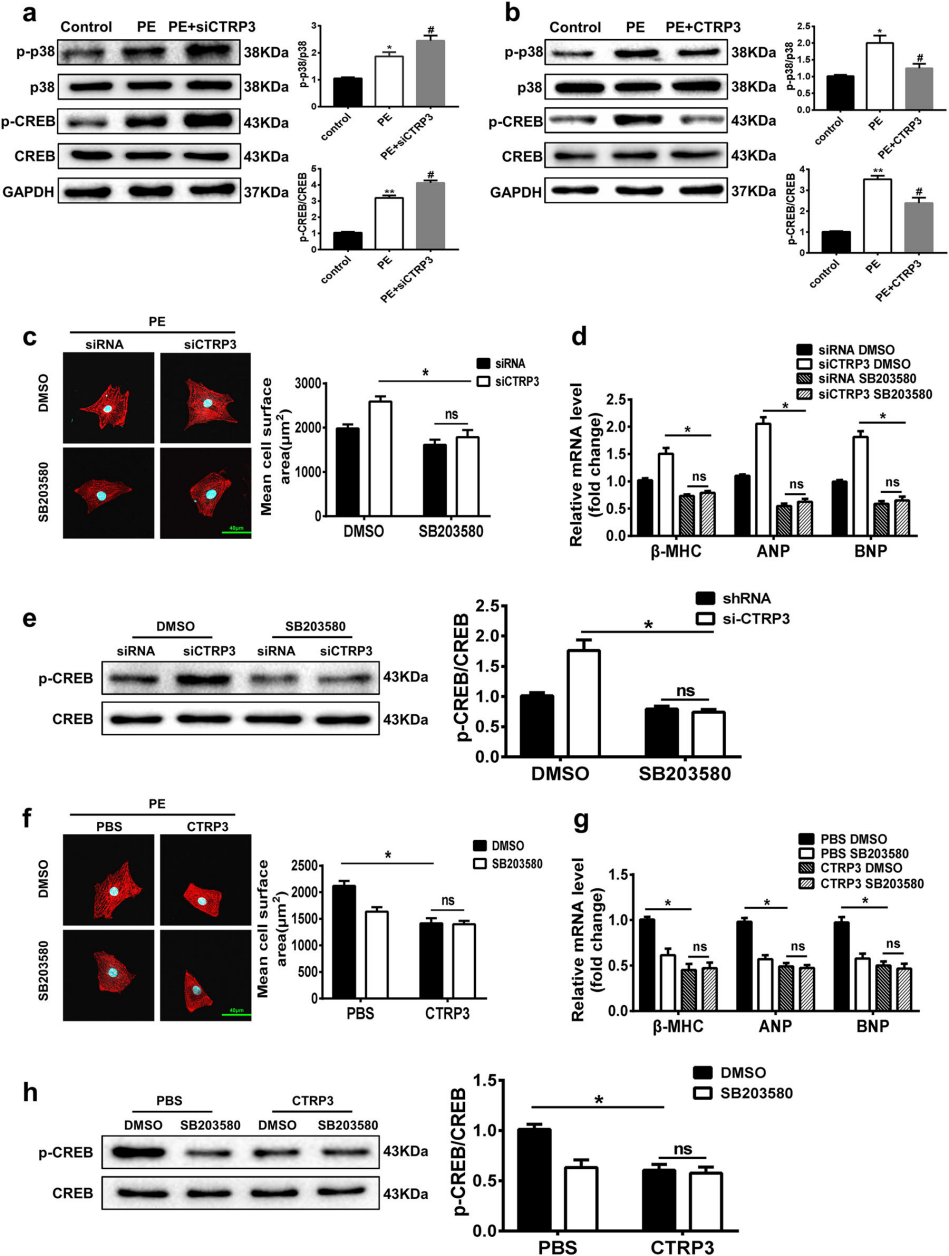
CTRP3 alleviates PE-induced cardiomyocyte hypertrophy in vitro by inhibiting the p38 MAPK pathway. **a** Representative western blot (left) and quantification (right) of the p38-CREB signaling pathway activity in NRCMs from the indicated groups (*n* = 5 samples per group). **b** Representative western blot (left) and quantification (right) of the p38-CREB signaling pathway activity in NRCMs from the indicated groups (*n* = 5 samples per group). **c** Left: Representative immunofluorescence images of NRCMs transfected with siRNA or si-CTRP3, and stained with α-actinin (red) and DAPI (blue). The NRCMs were treated with dimethyl sulfoxide (DMSO) or a p38 inhibitor SB203580 (1 μM). Right: The mean cell surface area of NRCMs in the indicated groups (*n* ≥ 30 cells per group). **d** Real-time PCR analysis of the expression of genes encoding the hypertrophic markers β-MHC, ANP, and BNP in the indicated groups (*n* = 5 samples per group). **e** Representative western blot (left), and quantification (right) of p-CREB and CREB levels in NRCMs from the indicated groups (*n* = 5 samples per group). **f** Left: Representative immunofluorescence images of PBS- and CTRP3-treated NRCMs stained with α-actinin (red) and DAPI (blue). The NRCMs were treated with DMSO or SB203580 (1 μM). Right: The mean cell surface area of NRCMs in the indicated groups (*n* ≥ 30 cells per group). **g** Real-time PCR analysis of the expression of genes encoding the hypertrophic markers β-MHC, ANP, and BNP in the indicated groups (*n* = 4 samples per group). **h** Representative western blot (left), and quantification (right) of p-CREB and CREB levels in NRCMs from the indicated groups (*n* = 5 samples per group). The data were analyzed by one-way ANOVA. **a**, and **b** *p < 0.05, **p < 0.01 vs. control, ^#^*p* < 0.05 vs. PE. **e**, **h** *p < 0.05 between the two indicated groups; ns, not significant. In the bar graphs, the data are presented as the mean ± SEM

We next proceeded to thoroughly investigate whether the p38/CREB signaling pathway participates in the antihypertrophic activity of CTRP3 in PE-induced cardiomyocyte hypertrophy. In PE-treated NRCMs, the phosphorylation level of p38 and CREB was increased, and si-CTRP3 administration exaggerated this response (Fig. 6a). However, CTRP3 supplementation inhibited the p38 and CREB activation induced by PE (Fig. 6b). Further, when p38 activity in PE-treated NRCMs was suppressed by SB203580, the PE-induced increase of cell size and expression of the hypertrophic genes were alleviated, indicating that phosphorylation of p38 was indeed involved in cardiomyocyte hypertrophy (Fig. 6c, d). In the presence of CTRP3 siRNA, the PE-induced increase of cell size and hypertrophic gene expression were exaggerated, while SB203580 blunted this effect (Fig. 6c, d). Similarly, CREB activation was inhibited by SB203580 (Fig. 6e). Finally, in the presence of CTRP3, the PE-induced increase of cell size and expression of the hypertrophic genes were alleviated, and SB203580 did not change this effect (Fig. 6f, g). CREB activation was also inhibited by CTRP3 and SB203580, but there was no significant change in the reduction of CREB expression when CTRP3 and SB203580 were provided simultaneously (Fig. 6h). Collectively, these findings indicate that CTRP3 inhibited PE-induced cardiomyocyte hypertrophy, at least partially, by inhibiting the activation of the p38/CREB signaling cascade.

### Protein p38-induced activation of the ER stress pathway is involved in pathological cardiac hypertrophy

**Fig. 7:**
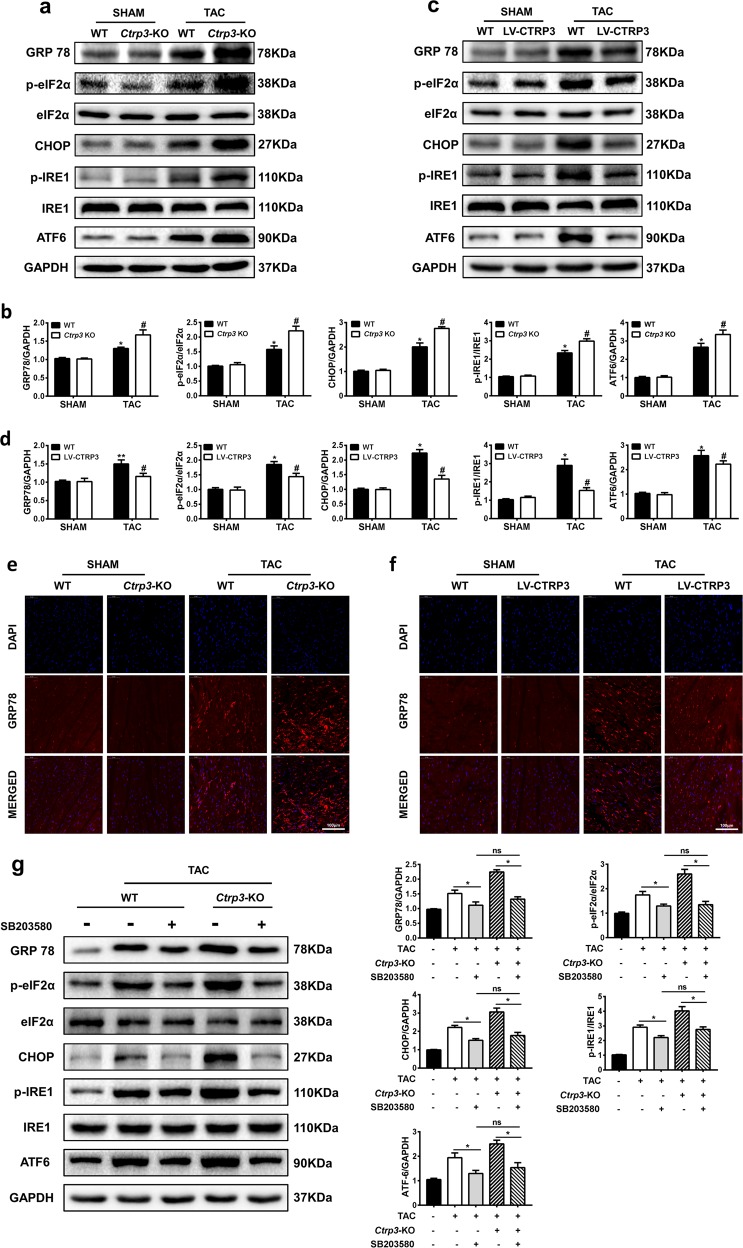
Protein p38-induced activation of the ER stress pathway is involved in pathological cardiac hypertrophy. **a** Representative western blot of the activity of GRP78, eIF2α, IRE1α, CHOP, and ATF6 in mice hearts from indicated groups. **b** Statistical diagrams of GRP78, eIF2α, IRE1α, CHOP, and ATF6 protein expression in mice hearts from indicated groups (*n* = 6 mice per group). **c** Representative western blot of the activity of GRP78, eIF2α, IRE1α, CHOP, and ATF6 in mice hearts from indicated groups. **d** Statistical diagrams of GRP78, eIF2α, IRE1α, CHOP, and ATF6 protein expression in mice hearts from indicated groups (*n* = 6 mice per group). **e, f** Representative immunofluorescence images of murine heart sections stained with GRP78 (red) and DAPI (blue) in the hearts of mice with different genotypes (WT, *Ctrp3*-KO, and LV-CTRP3) 4 weeks after sham treatment or TAC surgery (*n* = 5–6 mice per group). **g** Representative western blot (left), and quantification (right) of GRP78, eIF2α, IRE1α, CHOP, and ATF6 in the hearts of mice from the indicated groups (*n* = 5 mice per group). The data were analyzed by one-way ANOVA. **b**, **d** *p < 0.05, **p < 0.01 vs. SHAM, ^#^*p* < 0.05 vs. TAC. **g** *p < 0.05 and ^#^*p* < 0.05 between indicated groups. In the bar graphs, the data are presented as the mean ± SEM

In addition, we analyzed the protein levels of key ER stress molecules, such as GRP78 and the downstream signaling molecules eIF2α, CHOP, IRE1, and ATF6. CTRP3 deficiency exacerbated the TAC-induced activation of the ER stress signaling pathways (Fig. 7a–b). The immunofluorescence intensity of GRP78 was stronger in TAC-operated WT mice than in sham-operated WT mice, and CTRP3 deficiency enhanced the change (Fig. 7e). CTRP3 overexpression alleviated TAC-induced activation of the ER stress signaling pathways (Fig. 7c–f). We also examined the mRNA expression level of GRP78, ATF4, ATF6, and CHOP in vivo and in vitro experiments to further confirm the influence of CTRP3 on the ER stress signaling pathway. The results showed that TAC-induced upregulation of ER stress signaling mRNA levels were enhanced by CTRP3 deficiency and were attenuated by CTRP3 overexpression in vivo (Fig. S7a, b). In addition, the TAC-induced ER stress and ER stress after TAC exacerbated by *Ctrp3*-KO were alleviated by SB203580 and there is no significant difference between WT + TAC + SB203580 group and *Ctrp3*-KO + TAC + SB203580 group (Fig. 7g).

**Fig. 8:**
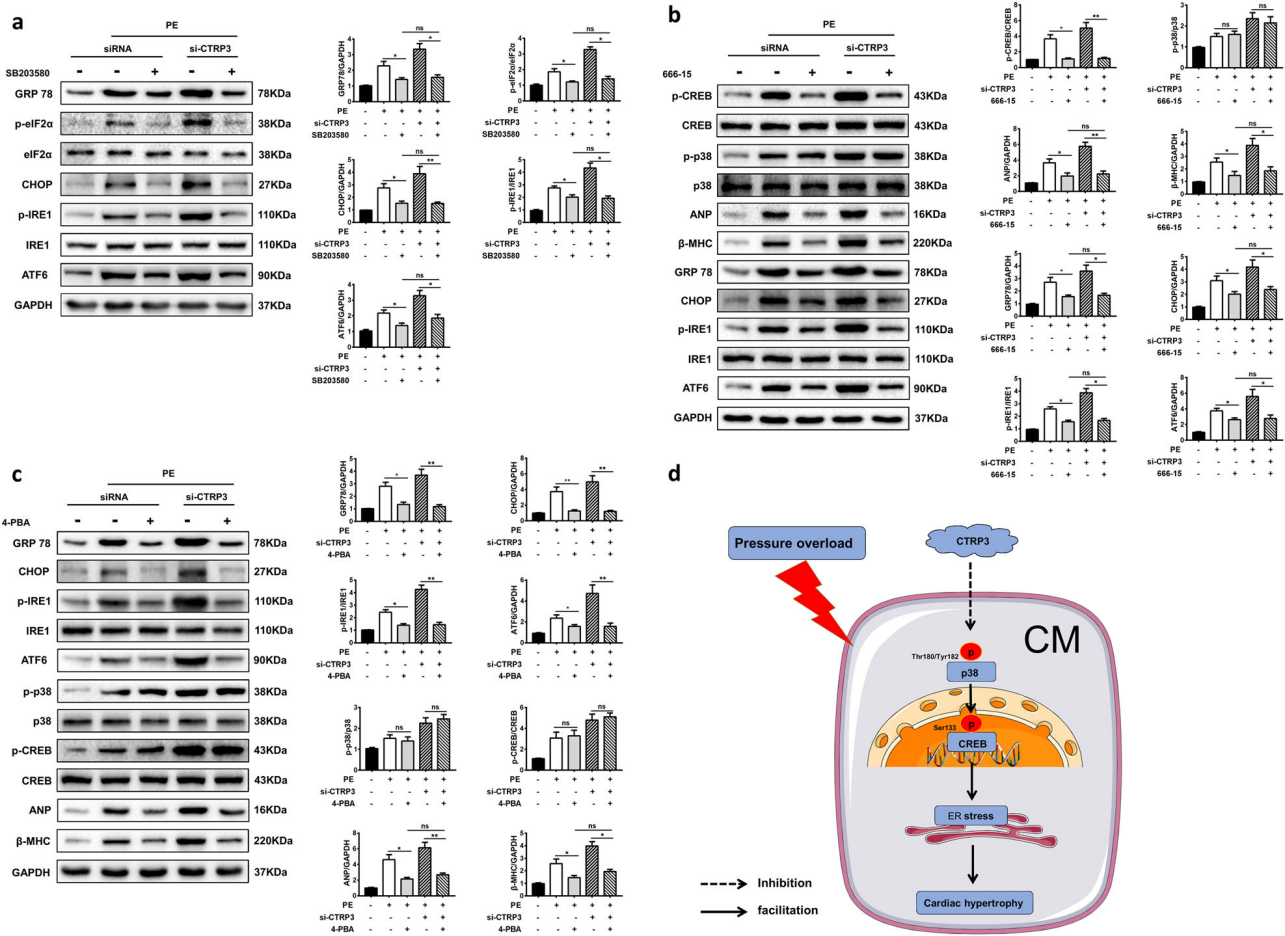
CTRP3 exerted its protective role on pathological cardiac hypertrophy through inhibiting p38/CREB/ER stress pathway. **a** Representative western blot (left) and quantification (right) of the ER stress signaling pathway activity in NRCMs from the indicated groups (*n* = 4 samples per group). **b** Representative western blot (left) and quantification (right) of the p38-CREB and ER stress signaling pathway activity in NRCMs from the indicated groups (*n* = 4 samples per group). **c** Representative western blot (left) and quantification (right) of ER stress and p38-CREB signaling pathway activity, protein expression levels of ANP and β-MHC in NRCMs from the indicated groups (*n* = 4 samples per group). *p < 0.05, **p < 0.01 between indicated groups. In the bar graphs, the data are presented as the mean ± SEM. **d** Proposed protective mechanism of the role of CTRP3 in pathological cardiac hypertrophy. CTRP3 inhibits the activation of the p38/CREB signaling pathway and ER stress pathway in cardiomyocytes under pressure overload, thereby alleviating pathological cardiac hypertrophy

In PE-treated NRCMs, the ER stress was also activated and exaggerated in the absence of CTRP3, while CTRP3 supplementation decreased the activation of ER stress (Fig. S8). Furthermore, SB203580 blunted the activation of ER stress induced by PE and attenuated the exaggerated ER stress in the presence of si-CTRP3, as evidenced by analysis of GRP78, IRE, eIF2α, and CHOP levels. There was no significant difference on activation of ER stress between PE + siRNA + SB203580 group and PE + si-CTRP3 + SB203580 group (Fig. 8a). Next, we pretreated NRCMs with 666–15 to inhibit activation of CREB. The results showed that CREB phosphorylation was effectively inhibited by 666–15. However, 666–15 made no obvious changes on PE-induced activation of p38. Moreover, 666–15 significantly decreased the protein expression of ANP and β-MHC and inhibited the activation of ER stress, including the expression of GRP78, CHOP, ATF6, and the phosphorylation of IRE1 in PE + siRNA + 666–15 group and PE + si-CTRP3 + 666–15 group. There was also no difference between the two groups (Fig. 8b). Finally, we treated NRCMs with 4-PBA to inhibit ER stress. We detected protein expression of GRP78, CHOP, and ATF6 and activation of IRE1 to confirm that PE-induced ER stress was inhibited. However, there is no significant change in activation levels of p38 and CREB. Furthermore, when p38 and CREB were activated to the same degree, inhibition of ER stress significantly reduced the protein expression of ANP and β-MHC in NRCMs from PE + siRNA + 4-PBA group and PE + si-CTRP3 + 4-PBA group. Moreover, there was no significant difference between the two groups (Fig. 8c). All these data indicated that ER stress acted as a downstream signaling pathway of p38/CREB in aggravated cardiac hypertrophy induced by CTRP3 deficiency.

Combined, these observations indicated that the suppression of p38-CREB and ER stress pathways involved in the protective effects of CTRP3 against pathological cardiac hypertrophy (Fig. 8d).

## Discussion

Since the 2017 ACC/AHA guidelines lowered the minimum level of hypertension to 130/80 mmHg, the number of adults with hypertension around the world further increased. Chronic hypertension caused long-term left ventricular pressure overload, leading to pathological cardiac hypertrophy and eventually heart failure^29^. It is wildly accepted that cardiac hypertrophy could act as an important therapeutic target for chronic heart failure^5^. But because the signaling pathway of cardiac hypertrophy is complex and imperfect, more basic and clinical studies are needed to explore the intervention strategies of cardiac hypertrophy^30,31^. In this study, we observed that CTRP3 deficiency aggravated the pressure overload-induced cardiac hypertrophy and fibrosis, while CTRP3 overexpression exerted a protective effect. We also found that CTRP3 exerted these effects by inhibiting the activation of p38-CREB and alleviating ER stress. The antihypertrophic effect of CTRP3 was verified in the in vitro PE-induced hypertrophic model.

Firstly, TAC surgery directly constricts the internal diameter of the aortic arch and increases the afterload of the left ventricle, and is widely used to research pressure-overload pathological cardiac hypertrophy and heart failure^32^. In this study, 4 weeks after TAC surgery, the heart function was weakened and myocardial fibrotic reconstruction formed. The impaired cardiac function and cardiac remodeling were deteriorated in *Ctrp3*-KO mice. However, these changes were attenuated by CTRP3 overexpression. After TAC, the expression of hypertrophic and fibrotic genes was significantly upregulated; CTRP3 deficiency enhanced this effect but CTRP3 overexpression mitigated it. The expression of α-MHC decreased and the expression of β-MHC increased in mice hearts after TAC. This conversion trend became accelerated in *Ctrp3*-KO mice but slowed down in LV-CTRP3 mice. A prolonged or severe pressure overload in the heart causes adaptive transformation of α-MHC to β-MHC^33^. During heart failure, the expression level of α-MHC gene decreases in ventricular myocytes^34^. Studies have confirmed that a continuous high expression of α-MHC exerts a certain protective effect on cardiac function^35^. The two major downstream regulatory proteins, CaN and phosphorylated-CaMKII, are closely associated with the development of myocardial hypertrophy^36^. Activation of CaN can regulate the expression of genes for hypertrophy-related molecules^37^. Inhibition of CaMKII activity can prevent cardiac hypertrophy^38,39^. In this study, changes in the CaN and p-CaMKII protein levels matched those of changes of other hypertrophic genes. We also used PE to induce cardiac hypertrophy in NCRMs^36^, and the results were consistent with those of in vivo experiments. Therefore, our results suggested an improved prognosis after TAC by CTRP3 overexpression.

Secondly, we revealed the role of the p38 MAPK/CREB signaling pathway in the anticardiac hypertrophy of CTRP3. In this study, the p-p38 and p-CREB were significantly upregulated in mice hearts after TAC, CTRP3 deficiency obviously exacerbated this effect, while CTRP3 overexpression suppressed it. Furthermore, the p38/CREB signaling pathway was confirmed to mediate give-and-take of CTRP3-regulated cardiac hypertrophy induced by TAC in vivo and PE in vitro. P38 MAPK is a stress-activated Ser/Thr kinase, activated by many pathological cardiovascular stressors, including hypertrophic stimuli^40^. Sustained activation of p38 contributes to myocyte hypertrophy and death in neonatal cardiomyocytes^41,42^. Further, multiple drugs and proteins protect against the pathological cardiac hypertrophy by inhibiting the p38 MAPK pathway^11,43^. CREB is a downstream effector of p38 MAPK^10,44^. The phosphorylated form of CREB reduces the level of Bcl-2 and enhances the level of the c-fos, mediating the occurrence of cardiac hypertrophy^45^. Several molecules can attenuate cardiomyocyte hypertrophy by inhibiting phosphorylation of CREB^16,46^.

Thirdly, we determined the role of p38-induced ER stress in the anticardiac hypertrophy of CTRP3. TAC-induced activation of ER stress including the activation of GRP78 and downstream signaling pathways, which was intensified in *Ctrp3*-KO mice but attenuated in LV-CTRP3 mice. In vitro experiments, CTRP3 knockdown aggravated ER stress induced by PE, while CTRP3 supplement significantly reduced it. In vitro studies proved that CTRP3 knockdown further activated p38, and enhanced ER stress in cardiomyocytes by activating downstream CREB, further aggravating the hypertrophy response of cardiomyocytes induced by PE. ER stress can be triggered by many pathological stimuli, including oxidative stress, ischemic insult, and disturbance in calcium homeostasis^18^. Recently, the involvement of ER stress in the many facets of cardiovascular disease was shown, including cardiac hypertrophy^19^. When unfolded proteins accumulate in the ER, GRP78 breaks the inactive state of three ER transmembrane sensors (PERK, IRE1, and ATF6) to reduce the unfolded protein level^47^. An obvious activation of the unfolded protein response is apparent both in heart failure patients and in TAC-operated mouse^20,48,49^. In TAC-operated mouse, CHOP plays a vital role in the pathological transition from cardiac hypertrophy to heart failure^20^. Furthermore, CHOP deficiency alleviates the cardiac hypertrophy in a mouse subjected to TAC^50^. All these studies suggest that ER stress is a critical molecular mechanism involved in cardiac hypertrophy. More importantly, p38 MAPK deficiency attenuates the activation of CHOP and cell apoptosis in a pressure-overloaded heart^51^. Further, in aging-induced cardiac malfunction, activated p38 MAPK mediates the activation of ER stress and cell apoptosis^52^, suggesting the regulating effect of p38 on ER stress. Our results confirmed that CTRP3 exerted a protective role in pathological cardiac hypertrophy at least through inhibiting the p38/CREB signaling pathway and alleviating the downstream ER stress. However, in the process of pathological cardiac hypertrophy, the direct molecule of CTRP3 in cardiac myocytes has not been clearly defined, and the downstream mechanism still needs to be further studied. Whether CTRP3 only delays the heart failure process, or can reverse pathological cardiac hypertrophy, further research is also needed.

According to our current study, we can explore a drug or agonist to intervene in patients with chronic hypertension, to a certain extent, activating the high expression of CTRP3 in myocardial tissue. CTRP3 overexpression can play a protective role in myocardium and delay the transition from cardiac hypertrophy to heart failure. Since CTRP3 supplement can also protect against PE-induced cardiomyocyte hypertrophy, direct exogenous supplementation of CTRP3 may also become an effective clinical therapeutic means, suggesting a broader clinical prospect.

Recently a study by Ma et al. showed different results that CTRP3 exacerbated cardiac hypertrophy in mice^31^. There are noteworthy differences about methods to abolish expression and overexpression of CTRP3 which may lead to different phenotypes. These results indicated that CTRP3 might exert different influences and mechanisms under different condition. Role and mechanism of CTRP3 in pathologic myocardial hypertrophy deserve our further studies.

In conclusion, CTRP3 protects the host against the TAC-induced cardiac hypertrophy and heart dysfunction by inhibiting the p38/CREB pathway and downstream ER stress. In vitro experiments similarly indicated that the stress-resistance role of CTRP3 in PE-induced cardiac hypertrophy was mediated by inhibiting the p38/CREB/ER stress pathway. These results provide new perspectives for the use of CTRP3 as a therapeutic target for treating pressure-overload cardiac hypertrophy and for preventing the transition from hypertrophy to heart failure. Further studies in thorough mechanisms involved are imperative before testing CTRP3 in clinical trials.

## Methods

### Experimental animals

All procedures involving the use and care of animals were performed according to the Guide for the Care and Use of Laboratory Animals of the Chinese Animal Welfare Committee and with the approval from the Fourth Military Medical University Committee on Animal Care. *Ctrp3*-KO mice were constructed by eliminating the second exon in *Ctrp3* gene (GenBank Accession No. NC_000081) using the clustered regularly interspaced short palindromic repeats (CRISPR)/Cas9 system at the Nanjing Biomedical Research Institute of Nanjing University (NBRI; Nanjing, China). *Ctrp3*-KO male mice and WT male C57BL/6 mice (20–25 g, 8–10-week-old, obtained from the Experimental Animal Center of the Fourth Military Medical University, Xi’an, Shaanxi, China), were housed in cages (10–12 animals/cage) under a 12:12-h light/dark cycle (lights on 06:00) at 22–24 °C, and had access to a regular pellet diet *ad libitum*. The surgery and subsequent analyses were performed as specified below.

### Preparation and intramyocardial injection of the lentivirus (LV)-CTRP3 construct

Recombinant lentivirus overexpressing mouse *Ctrp3* (NM_001204134) was constructed by Shanghai Genechem Co., Ltd (Shanghai, China). WT male C57BL/6 mice (6–8 weeks old) were anesthetized in an induction chamber with 2% (v/v) isoflurane mixed with pure oxygen (0.5–1.0 l/min). Intramyocardial injection was performed as follows^53^. An oblique incision ~0.5-cm long was made between the fourth and fifth costal margin of the left side of the sternum. The heart was then squeezed by exerting pressure on the right chest wall. The lentivirus was then provided by an intramuscular injection from the front, side, and back of the left ventricle, the total amount is 25 μl (5 × 10^6^ drips of CTRP3-overexpressing lentivirus). LV-NULL was injected in a similar manner into another group of mice. The heart was then repositioned in the chest cavity and the chest wall sutured. Sham and TAC surgeries were performed 2 weeks later.

### Expression and production of CTRP3

CTRP3 was overproduced and purified as previously reported^22^. Briefly, the *Ctrp3* gene (GenBank Accession No. NM_030945.3) was synthesized by GENEWIZ (Jiangsu, China) and cloned into the prokaryotic protein expression vector pET30a (Novagen, Merck Eurolab, Fontenay-sous-Bois, France). The expression vector was then transferred into *Escherichia coli* BL21(DE3) for protein production. BL21(DE3)/pET30a-CTRP3 were grown in LB medium (NaCl 10 g/L, tryptone 10 g/l, yeast extract 5 g/l), with shaking at 37 °C for 3 h. Isopropyl-β-d-thiogalactoside was then added to the medium (final concentration, 1 mM). The solution was shaken overnight at 16 °C and then centrifuged at 5000 × *g*. Proteins were purified under native conditions on a HisTrap HP column (GE Healthcare, Madison, Wisconsin, USA), as per the manufacturer’s instructions. Endotoxin was removed by passing through an endotoxin-removal column (high-capacity endotoxin removal spin column, Pierce, Thermo Fisher Scientific, San Jose, California, USA). The purified protein was then desalted and concentrated by centrifugation using Amicon ultra-15 centrifugal filter units (UFC900396, Millipore, Billerica, MA, USA).

### TAC

The murine chronic cardiac hypertrophy model was established by TAC surgery. TAC surgery was performed as previously described^54^. Briefly, C57BL/6 mice were anesthetized in an induction chamber with 2% isoflurane mixed with pure oxygen (0.5–1.0 l/min). The animals were orally intubated with a 20-gauge tube and ventilated (Minivent Type 845, Hugo Sachs Electronic, March, Germany) at 100–120 times per minute (0.15-ml tidal volume). A median thoracotomy was performed at the second intercostal space. The transverse aorta was constricted by using a 7–0 silk suture ligature tied firmly against a 27-gauge needle between the carotid arteries. The needle was then promptly removed. Sham-operated mice underwent an identical surgical procedure except for the ligation of the aorta. The chest was closed with a 6–0 silk suture and the skin was closed with a 5–0 silk suture. After TAC, the mice were kept warm on a 38 °C constant temperature plate and carefully monitored until free to move.

### Experimental design and treatment

Fifteen WT mice were randomly selected as the SHAM group. Twelve WT mice and twelve *Ctrp3*-KO mice were randomly selected as the SHAM + LV-CTRP3 group and SHAM + CTRP3-KO group, respectively. Fifteen SHAM mice were selected for intramyocardial injection of LV-NULL, and twelve SHAM + LV-CTRP3 mice were given intramyocardial injection of LV-CTRP3. Two weeks later, all the above mice underwent sham surgery.

Fifteen WT mice were randomly selected as the TAC group. Twelve WT mice and twelve *Ctrp3*-KO mice were randomly selected as the TAC + LV-CTRP3 group and TAC + CTRP3-KO group, respectively. Fifteen TAC mice were selected for intramyocardial injection of LV-NULL, and twelve TAC + LV-CTRP3 mice were given intramyocardial injection of LV-CTRP3. Two weeks later, all the above mice underwent TAC surgery.

Thirty WT mice were randomly selected and separately divided into TAC + SB203580, TAC + LV-CTRP3, and TAC + LV-CTRP3 + SB203580 groups (10 mice/group). Twenty *Ctrp3*-KO mice were randomly selected, and separately arranged into TAC + CTRP3-KO group and TAC + CTRP3-KO + SB203580 groups (10 mice/group). SB203580 (a p38 MAPK inhibitor, purchased from Calbiochem (La Jolla, CA, USA)) was injected intraperitoneally into mice from the indicated groups every day after TAC operation. To do this, SB203580 was dissolved in 1% DMSO (1 mg/ml). The mice were then injected intraperitoneally with SB203580 (10 mg/kg)^55^. The remaining mice were injected with the same amount of 1% DMSO.

### NRCM culture

NRCMs were obtained from the ventricles of newly born Sprague Dawley (SD) rats (male, obtained from the Experimental Animal Center of the Fourth Military Medical University, Xi’an, Shaanxi, China), as described previously^56^. Briefly, newly born SD rats were disinfected twice with 75% alcohol, the heart was harvest and then cut up in serum-free Dulbecco’s Modified Eagle’s Medium/Nutrient Mixture F-12 Ham (DME/F-12, 1:1 mixture; Gibco, Carlsbad, CA, USA). Then, the tissue fragments were digested in PBS solution containing 1% collagenase-I (Sigma V900891; Sigma-Aldrich, St. Louis, MO, USA), three times, 5 min each time. Cell suspension was then placed in DME/F-12 containing 10% serum, centrifuged for 5 min at 1000 rpm, and the supernatant was discarded. The cells were resuspended in the culture medium containing serum [DME/F-12, 10% new bovine serum (Gibco, Carlsbad, CA, USA), penicillin (100 U/ml), streptomycin (100 U/ml), and bromodeoxyuridine (BrdU) (0.1 mM; to inhibit fibroblast proliferation)], and placed in a culture box for 1–1.5 h, using the differential velocity adherent method to purify the myocardial cells. The myocytes were plated in 6-well plates or confocal dishes at a density of 5 × 10^5^ cells/ml and cultured for 24 h at 37 °C with 5% CO_2_. The NRCMs were then incubated with PE (50 μM) for 24 h to induce cardiomyocyte hypertrophy. Successful induction of the cardiomyocytes was determined by measuring the cell surface area (after α-actinin staining) and hypertrophy-related gene expression levels (β-MHC and ANP). A part of NRCMs were firstly transfected with si-CTRP3 using Lipofectamine 2000^27^ (Thermo Fisher Scientific, San Jose, California, USA) before PE treatment. Some NRCMs were also treated with CTRP3 (5 μg/ml)^22^ in the presence of PE for 24 h. Before PE treatment, some NRCMs were pretreated respectively with 1 μM SB203580 or 1 μM 666–15 (CREB inhibitor, purchased from Med Chem Express) for 2 h to inhibit phosphorylation of p38 or CREB^57^. In order to inhibit ER stress, some NRCMs were treated with 2 mM 4-PBA (ER stress inhibitor, purchased from Sigma-Aldrich, St Louis, MO, USA) in the presence or absence of PE for 24 h^58^. The NRCMs after PE treatment were subsequently prepared for use in other experiments as described below.

### Echocardiography

The echocardiographer was blinded to the experimental protocols and surgical procedure. Before measuring the echocardiography, mouse was anesthetized in an induction chamber with 2% isoflurane mixed with pure oxygen (0.5–1.0 l/min). The thermostatic plate was adjusted to 42 ℃ and the mouse body temperature was maintained at about 38 ℃. The heart rate of the mouse was maintained at 400–500 beats per minute by adjusting the concentration of isoflurane. Transthoracic ultrasonography was performed using a VisualSonics 770 echocardiograph (VisualSonics 770, Toronto, ON, Canada), and a 30-MHz transducer was used to record the views in both parasternal long-axis and short-axis of the left ventricle. LVEF was used to represent the left ventricular contractile function. IVSd and LVPWd were determined as indicators of cardiac hypertrophy. The above parameters were measured and calculated by Vevo Lab 3.1.0 software (FUJIFILM VisualSonics, Inc. Toronto, ON, Canada).

### Histological analysis

Four weeks after the sham or TAC surgery, 5–6 mice from each group were weighed and then euthanized by carotid artery bleeding under anesthesia. The heart was exposed by shear, the aorta was occluded, and incision was made in the inferior vena cava. The heart was perfused by 1 ml syringe with phosphate-buffered saline (PBS) to flush the blood out of the heart via the cardiac apex, twice, and was then perfused with 4% paraformaldehyde. The heart was then harvested and fixed in 4% paraformaldehyde in a 1.5-ml tube at 4 °C for at least 24 h. It was cut into 5-μm-thick sections from the cardiac apex, and the sections were stained with HE, WGA, and Masson trichrome stain to evaluate the cross-sectional area of the cardiomyocytes and the degree of fibrosis. The sections were visualized by a digital scanning imaging system Olympus FV1000 (Olympus, Tokyo, Japan) and the cross-sectional area of the cardiomyocytes and degree of fibrosis were quantified by using Image J software (NIH, Bethesda, MD, USA).

### Quantitative real-time PCR

Mice from each group other than the ones used for histological analysis were weighed and then euthanized as mentioned above. The hearts were harvested and washed with PBS; HW was then recorded. The HW/BW ratio was then calculated. A small portion was cut off from the left ventricular wall and placed in a 1.5-ml tube containing 1 ml Trizol reagent (TIANGEN, Beijing, China). Total RNA was extracted from the left ventricular tissue specimen (and the cultured NRCMs) according to the protocol of the total RNA extraction kit (TIANGEN, Beijing, China). The RNA was then reverse-transcribed into complementary DNA. The CFX96 real-time PCR system C1000 Thermal Cycler (Bio-Rad Laboratories, Hercules, CA, USA) was used for real-time PCR. *GAPDH* served as the standard gene for the normalization of transcript levels of target genes. The primers used in this study were synthesized by GenScript Biotech Corp. (Nanjing, China) and the sequences are listed in Table S1.

### Western blotting

Total protein from the mouse left ventricular wall and interventricular septum, and from the treated NRCMs was extracted in the RIPA lysis buffer^56^. The protein concentration in samples was determined using the bicinchoninic acid assay (Solarbio co, LTD, Shanghai, China). Extracted protein was separated by 10% sodium dodecyl sulfate-polyacrylamide gel electrophoresis (SDS-PAGE). Then, proteins from the SDS-PAGE gel were transferred onto a polyvinylidene fluoride membrane (Millipore, Billerica, MA, USA). Membranes were blocked in 5% skim milk powder and dissolved in TBST buffer [150 mM NaCl, 50 mM Tris (pH 7.5), and 0.1% Tween-20) for 2–3 h at 20–25 °C. The membranes were then cut horizontally and incubated with the respective primary antibody overnight at 4 °C. The membrane strips were washed with TBST and then incubated with the secondary antibody conjugated with horseradish peroxidase for 2 h at 20–25 °C. The ECL reagent (Millipore, Billerica, MA, USA) was added and the blots were scanned using ChemiDoc™ XRS (Bio-Rad Laboratories, Hercules, CA, USA). The gray value of protein bands was determined using Image Lab 2.0(Genmall Biotechnology Co.,Ltd, Wuhan, China) and GADPH was used as the internal control. The primary antibodies used were anti-p-CREB (Ser133; 9198 s), anti-CREB (9197 s), anti-p-CaMKII (12716), anti-p-eIF2α (3398), anti-eIF2α (5324), anti-ATF4 (11815), anti-CHOP (2895), anti-p-AMPKα (2537), anti-AMPKα (5832), anti-p-AKT (4060), anti-AKT (9272), anti-Bcl-2 (3498), and anti-Bax (5023) antibodies, purchased from Cell Signaling Technology (Beverly, MA, USA); anti-ANP (ab209232), anti-CaN A (ab3673), anti-CaMK□ (ab52476), anti-GRP78 (ab21685), anti-p-IRE1 (ab48187), anti-IRE1 (ab37073), anti-XBP1 (ab37152), anti-ATF6 (ab203119), and anti-CTRP3 (ab36870) antibodies, purchased from Abcam (Cambridge, MA, USA); anti-p-p38 (sc-166182), anti-p38 (sc-7149), and anti-MYH7 (sc-53089) antibodies, purchased from Santa Cruz Biotechnology (Santa Cruz, CA, China); and anti-GAPDH (AT0002) antibody purchased from cmcTAG (Milwaukee, WI, USA). The secondary antibodies used were goat anti-rabbit (ZB-2301) and goat anti-mouse (ZB-2305) antibodies purchased from Zhongshan Company (Beijing, China).

### Immunostaining

Immunofluorescence staining was performed as described previously^59^. First, the expression and activation of p38 and GRP78 were detected by immunofluorescence. Briefly, sections were permeabilized in 0.05% Triton X-100 for 10 min. They were washed three times with PBS and incubated with 1% bovine serum albumin for 1 h at room temperature. The sections were incubated with mouse anti-p-p38 antibody or rabbit anti-GRP78 antibody (details in western blot chapter, both 1:50 dilution) for 10–12 h at 4 °C. Antigen-antibody complexes were visualized by goat anti-mouse or anti-rabbit IgG antibodies were conjugated with Alexa Fluor^®^Cy3. Nuclei were visualized by DAPI. The cardiomyocytes were then counted. The p-p38 MAPK activity was expressed as the average percentage of nuclei positive for p-p38 MAPK per section.

Immunofluorescence staining was also used to determine the surface area of cardiac myocytes in each animal group. After treatment, confocal dish containing NRCMs was washed with PBS, three times, and then fixed with 4% paraformaldehyde at 4 °C for 30 min. After washing three times with PBS, the cells were permeabilized with 0.05% Triton X-100 for 10 min, and then washed again three times with PBS. Next, they were incubated with 1% bovine serum albumin for 30 min at room temperature, followed by incubations with α-actinin (Sigma A7732, Sigma-Aldrich, St. Louis, MO, USA) for 10–12 h 4 °C. After incubating with a secondary antibody labeled with horseradish peroxidase at 37 °C for 2 h, the cells were washed three times with PBS. The nucleus was stained with DAPI (purchased from BIO-LIEESCICO.,LTD, Guangzhou, China) and the cells were washed three times with PBS. The cells were then observed under a confocal microscope. For each group, at least 50 cells were analyzed at random. The Image J software (NIH) was used to calculate the cardiomyocyte surface area.

### ELISA

Mouse carotid blood was collected by carotid artery bleeding. After 6 h, 1 ml of the whole blood was centrifuged at 3000 rpm for 10 min. The upper serum layer was aspirated into a 1.5-ml centrifugation tube and the serum CTRP3 level was detected by ELISA (SEK169Mu, CLOUD-CLONE CORP., Wuhan, China). All sample handling was performed according to the instructions of the ELISA kit manufacturer.

### Statistical analysis

All generated data were processed and analyzed using GraphPad Prism 7.0 (GraphPad Software, In., San Diego, CA, USA). In all bar graphs, the data are presented as the mean ± SEM. The differences between two groups were analyzed by the *t*-test. Statistical differences between multiple groups were analyzed by one-way ANOVA. In this study, *p* < 0.05 represents a statistically significant difference.

## Supplementary information

supplement material
