# Supplementary material for: C1q-TNF-related protein-3 attenuates pressure overload-induced cardiac hypertrophy by suppressing the p38/CREB pathway and p38-induced ER stress
Source: Cell Death Dis. 2019 Jul 8;10(7):520. doi: 10.1038/s41419-019-1749-0 (PMC6614451; doi:10.1038/s41419-019-1749-0)
Supplement: Supplementary file 1 — supplement material [file 41419_2019_1749_MOESM1_ESM.docx]

**Supplementary tables and figures**

**Table.S1 A list of the primers used in the RT- PCR**

| **Gene** | **Forward primer** | **Reverse primer** |
| --- | --- | --- |
| GAPDH（mouse） | AGAACATCATCCCTGCATCC | AGTTGCTGTTGAAGTCGC |
| α-MHC（mouse） | TGCACTACGGAAACATGAAGTT | CGATGGAATAGTACACTTGCTGT |
| β-MHC（mouse） | ACTGTCAACACTAAGAGGGTCA | TTGGATGATTTGATCTTCCAGGG |
| ANP（mouse） | TCTTCCTCGTCTTGGCCTTT | CCAGGTGGTCTAGCAGGTTC |
| BNP（mouse） | TGGGAGGTCACTCCTATCCT | GGCCATTTCCTCCGACTTT |
| Acta-1（mouse） | CCAAAGCTAACCGGGAGAAG | GACAGCACCGCCTGGATAG |
| IL-6（mouse） | CTGCAAGAGACTTCCATCCAG | AGTGGTATAGACAGGTCTGTTGG |
| Rcan1.4（mouse） | TTGTGTGGCAAACGATGATGT | CCCAGGAACTCGGTCTTGT |
| TGF-β（mouse） | GAGCCCGAAGCGGACTACTA | TGGTTTTCTCATAGATGGCGTTG |
| Collagen-1（mouse） | CTGGCGGTTCAGGTCCAAT | TTCCAGGCAATCCACGAGC |
| Collagen-3（mouse） | TGAATGGTGGTTTTCAGTTCAG | GATCCCATCAGCTTCAGAGACT |
| GRP78（mouse） | AGGAGGACAAGAAGGAGGA | GAGTGAAGGCCACATACGA |
| ATF4（mouse） | ACTTCGATGCTCTGTTTCG | GCTCATCTGGCATGGTTT |
| ATF6（mouse） | CTGGCAAAGCAGCAGTC | GGCAGCACATTCACCAC |
| CHOP（mouse） | GAACAGTGGGCATCACCTC | CAGTCCCCTCCTCAGCAT |
| GAPDH（rat） | TGACAACTCCCTCAAGATTGTCA | GGCATGGACTGTGGTCATGA |
| β-MHC（rat） | CAGGCCAACACCAACCTGTC | TCTACTCTTCATTCAGGCCCTTGG |
| ANP（rat） | CTGGGACCCCTCCGATAGAT | TTCGGTACCGGAAGCTGTTG |
| BNP（rat） | CAATCCACGATGCAGAAGCTG | GGCGCTGTCTTGAGACCTAA |
| GRP78（rat） | AGGAGGACAAGAAGGAGGA | GAGTGAAGGCCACATACGA |
| ATF4（rat） | TGAACAGCGAAGTGTTGG | TGAGGTTTGAAGTGCTTGG |
| ATF6（rat） | CAAGCGGAGAACCACCT | CACGGCCATCAGAGAACT |
| CHOP（rat） | GACTTGACCCGCCTCTC | TGGTCTCTCCTGGCCTAA |
| CTRP3（rat） | CATGGAGGTGAGCAGAAGA | ACAGTCCCCGTTTTAGCA |

**Fig.S1**





**Fig. S1. a** Representative western-blot bands (left) and statistical diagrams (right) of CTRP3, ANP and β-MHC protein expression in mice hearts after TAC (n=5 mice per group). **b** Statistical diagram of CTRP3 mRNA expression in mice hearts after TAC (n=5 mice per group). **c** Representative western-blot bands (left) and statistical diagrams (right) of CTRP3, ANP and β-MHC protein expression in NRCMs after PE treatment (n=4 samples per group). **d** Statistical diagram of CTRP3 mRNA expression in NRCMs after PE treatment (n=4 samples per group). The data were processed in t-test. *p<0.05, **p<0.01 vs. SHAM or Control. The column graphs were made as mean±SEM of the data.

**Fig.S2**





**Fig. S2. a** LW/BW ratio calculated with the weights of body and heart of mice from indicated groups after 4 weeks of TAC surgery (n=7 mice per group). **b** FS% analysed via measuring the echocardiographic images of mice from indicated groups (n=10-12 mice per group). **c** LW/BW ratio calculated with the weights of body and heart of mice from indicated groups after 4 weeks of TAC surgery (n=6-7 mice per group). **d** FS% analysed via measuring the echocardiographic images of mice from indicated groups (n=10-12 mice per group). The data were processed in one-way ANOVA. *p<0.05 vs. SHAM, ^#^p<0.05 vs. TAC. The column graphs were made as mean±SEM of the data.

**Fig.S3**


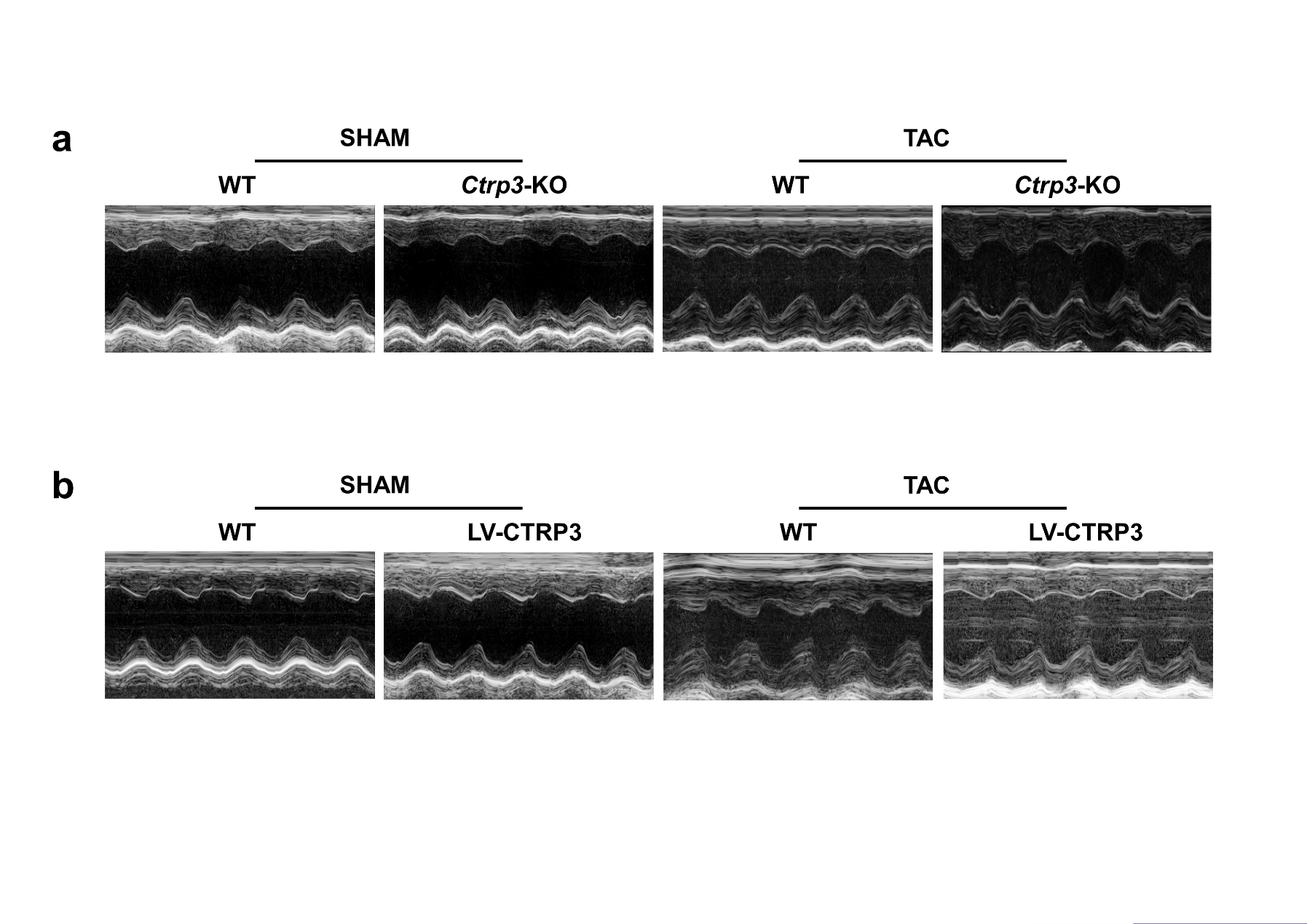


**Fig. S3. a b** representative M-mode echocardiographic images of the indicated groups (n=10-12 mice per group).

**Fig.S4**


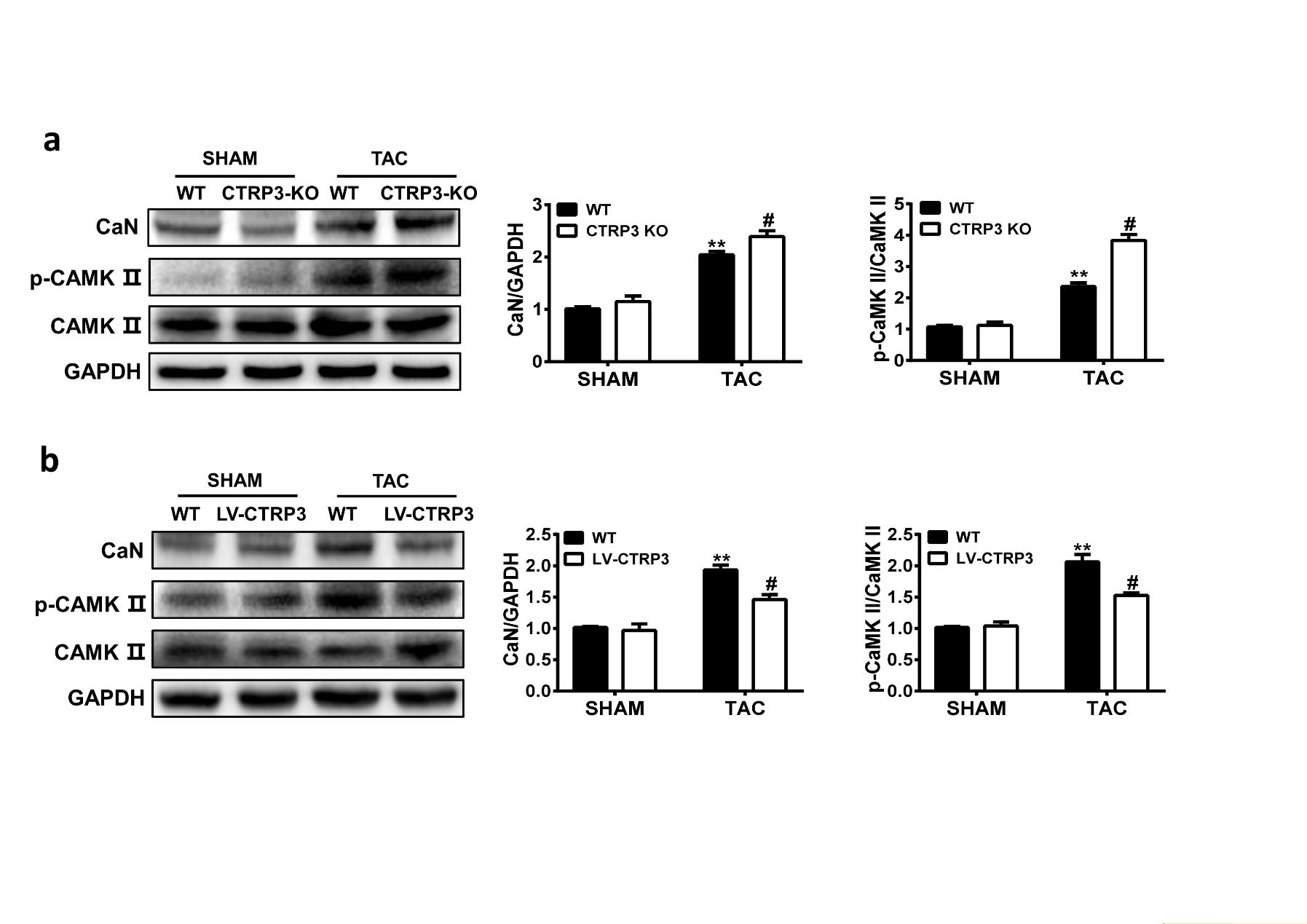


**Fig. S4. a b** Representative western-blot bands **(left)** and statistical diagrams **(right)** of calcineurin (CaN) and calmodulin kinase Ⅱ (CaMKII) activation in mice hearts from the indicated groups(n=5-6 mice per group). The data were processed in one-way ANOVA. *p<0.05, **p<0.01 vs. SHAM, #p<0.05 vs. TAC. The column graphs were made as mean±SEM of the data.

**Fig.S5**


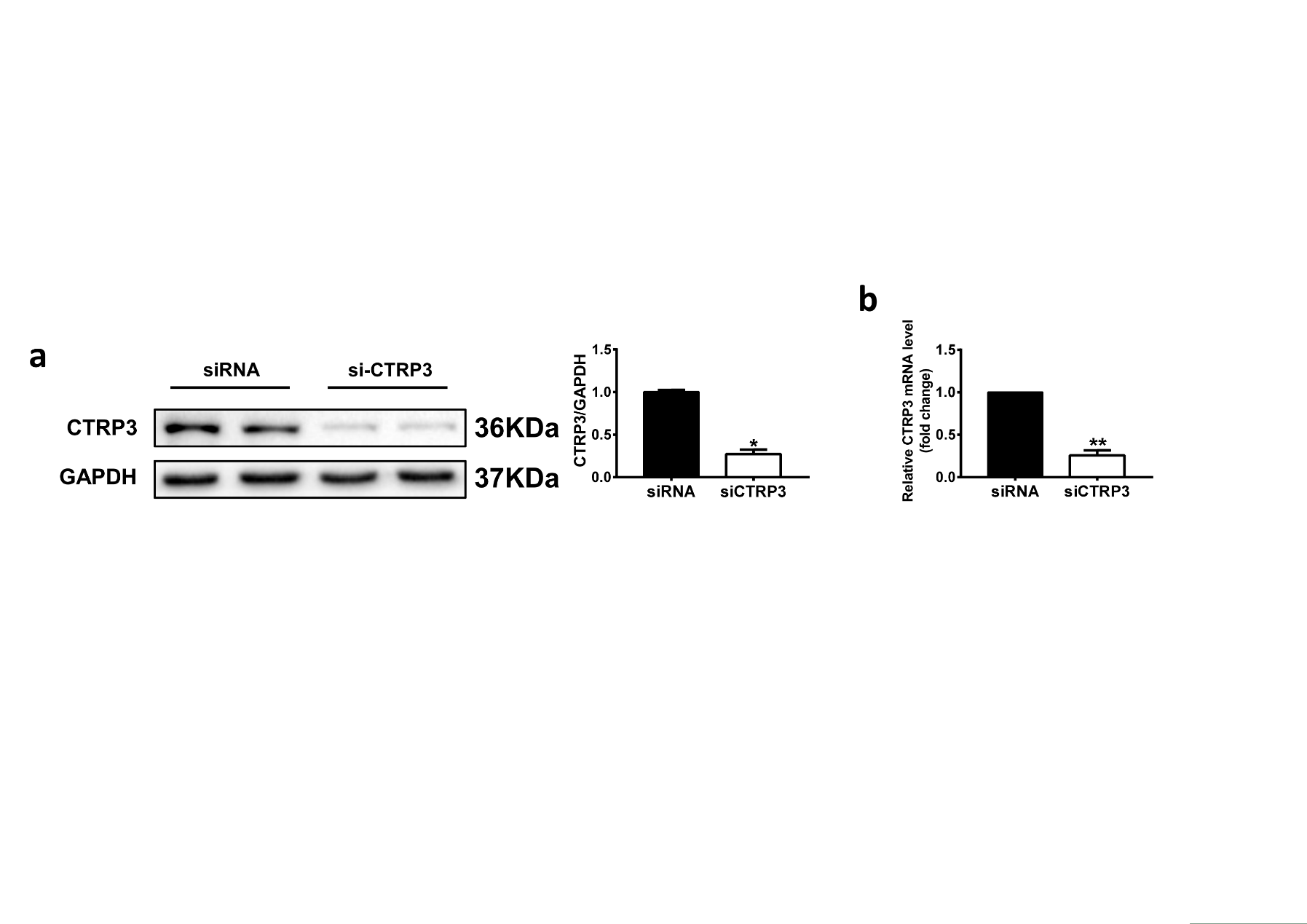


**Fig. S5. a** Representative western-blot bands (left) and statistical diagrams (right) of CTRP3 protein expression in NRCMs after si-CTRP3 transfection (n=4 samples per group). **b** Statistical diagram of CTRP3 mRNA expression in NRCMs after si-CTRP3 transfection (n=4 samples per group). The data were processed in t-test. *p<0.05, **p<0.01 vs. siRNA. The column graphs were made as mean±SEM of the data.

**Fig.S6**


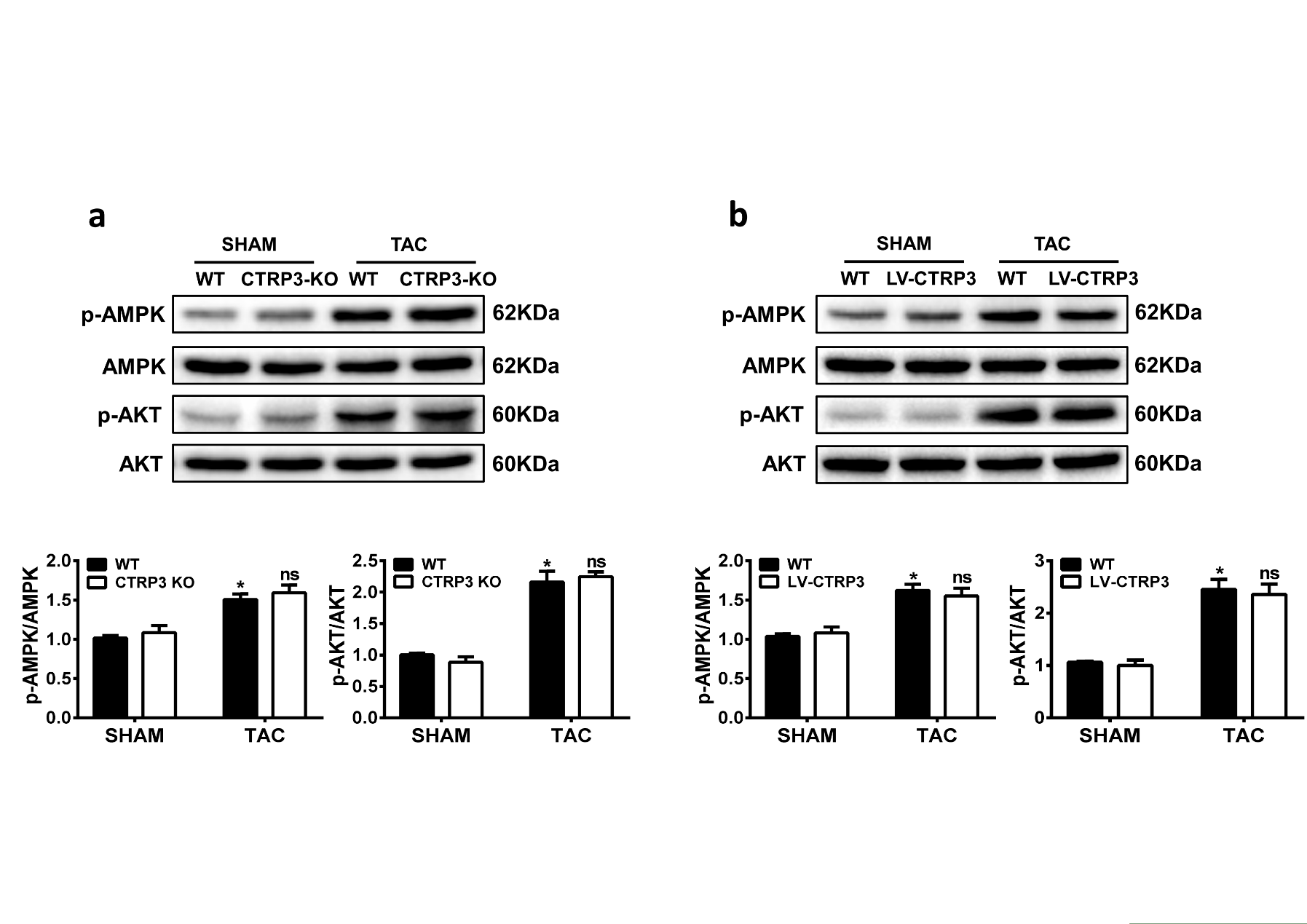


**Fig. S6. a b** Representative western-blot bands (top) and statistical diagrams (bottom) of AMPK and AKT activation in mice hearts from the indicated groups(n=5-6 mice per group). The data were processed in one-way ANOVA. *p<0.05 vs. SHAM, ^ns^ p>0.05 vs. TAC. The column graphs were made as mean±SEM of the data.

**Fig.S7**


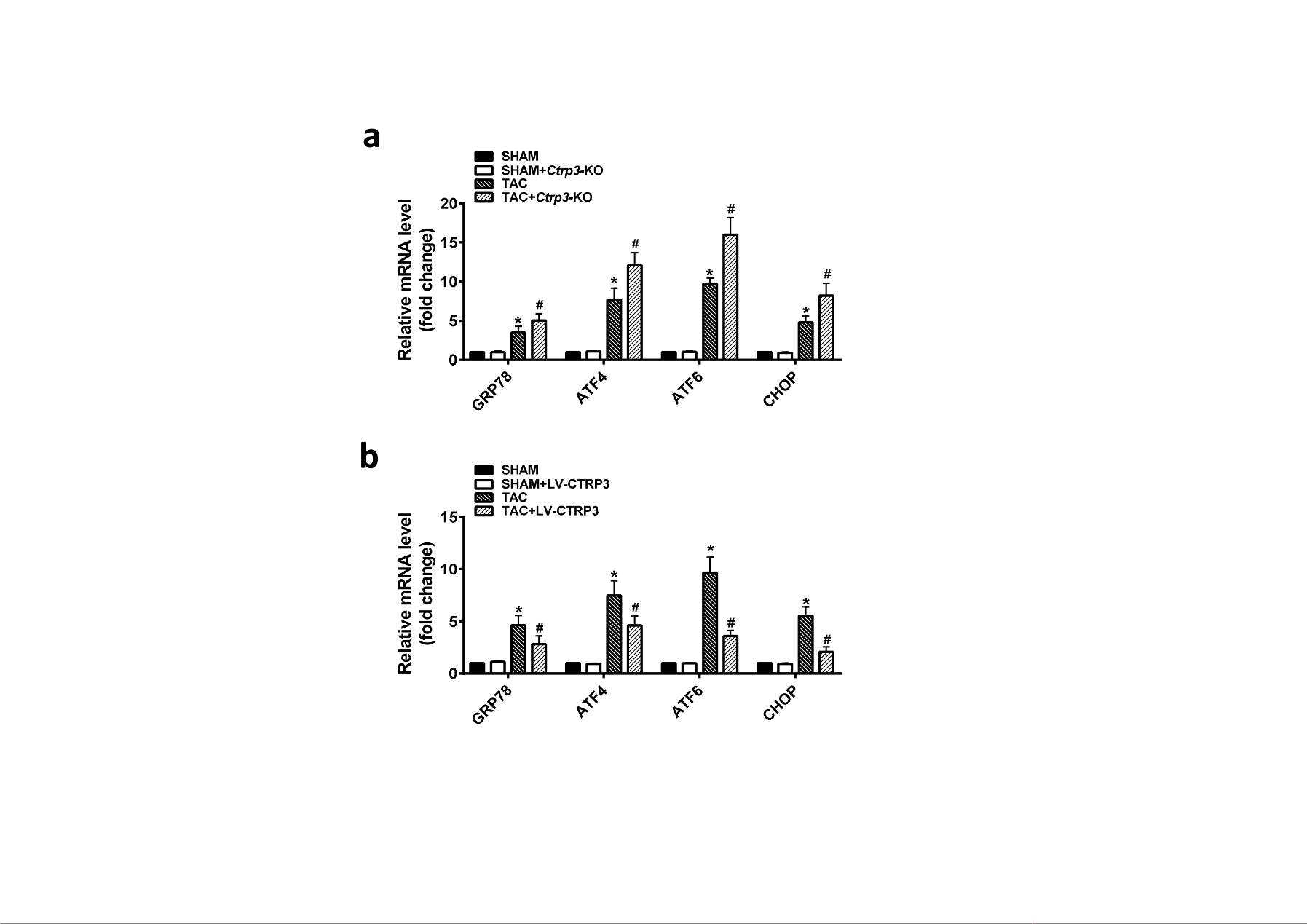


**Fig. S7. a b** Statistical diagram of ER stress markers mRNA expression in mice hearts from indicated groups (n=6 mice per group). The data were processed in one-way ANOVA. *p<0.05 vs. SHAM, ^#^p<0.05 vs. TAC. The column graphs were made as mean±SEM of the data.

**Fig. S8**


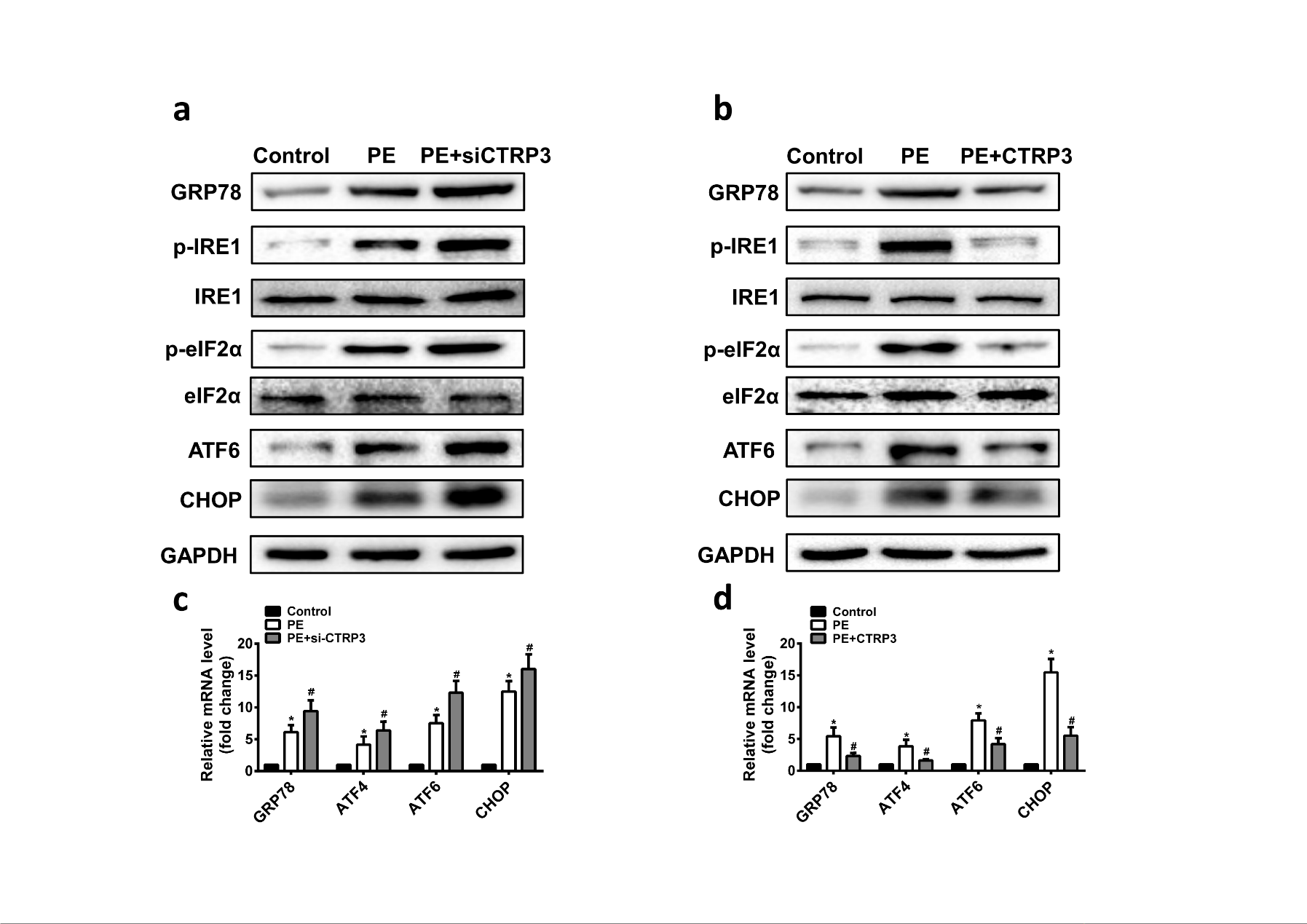


**Fig. S8. a** **b** Representative western-blot bands of activity of GRP78, eIF2α, IRE1α and CHOP in NRCMs from indicated groups. (n=4 samples per group) **c d** Statistical diagram of ER stress markers mRNA expression in NRCMs from indicated groups (n=4 samples per group). The data were processed in one-way ANOVA. *p<0.05 vs. Control, ^#^p<0.05 vs. PE. The column graphs were made as mean±SEM of the data
